# Minimizing the *Ex Vivo* Confounds of Cell-Isolation Techniques on Transcriptomic and Translatomic Profiles of Purified Microglia

**DOI:** 10.1523/ENEURO.0348-21.2022

**Published:** 2022-03-28

**Authors:** Sarah R. Ocañas, Kevin D. Pham, Harris E. Blankenship, Adeline H. Machalinski, Ana J. Chucair-Elliott, Willard M. Freeman

**Affiliations:** 1 Genes & Human Disease Program, Oklahoma Medical Research Foundation, Oklahoma City, OK 73104; 2 Department of Physiology, University of Oklahoma Health Sciences Center, Oklahoma City, OK 73104; 3 Oklahoma City Veterans Affairs Medical Center, Oklahoma City, OK 73104; 4 Department of Biochemistry and Molecular Biology, University of Oklahoma Health Sciences Center, Oklahoma City, OK 73104

**Keywords:** brain, cell sorting, *ex vivo* activation, methods, microglia, transcriptome

## Abstract

Modern molecular and biochemical neuroscience studies require analysis of specific cellular populations derived from brain tissue samples to disambiguate cell type-specific events. This is particularly true in the analysis of minority glial populations in the brain, such as microglia, which may be obscured in whole tissue analyses. Microglia have central functions in development, aging, and neurodegeneration and are a current focus of neuroscience research. A long-standing concern for glial biologists using *in vivo* models is whether cell isolation from CNS tissue could introduce *ex vivo* artifacts in microglia, which respond quickly to changes in the environment. Mouse microglia were purified by magnetic-activated cell sorting (MACS), as well as cytometer-based and cartridge-based fluorescence-activated cell sorting (FACS) approaches to compare and contrast performance. The Cx3cr1-NuTRAP mouse model was used to provide an endogenous fluorescent microglial marker and a microglial-specific translatome profile as a baseline comparison lacking cell isolation artifacts. All sorting methods performed similarly for microglial purity with main differences being in cell yield and time of isolation. *Ex vivo* activation signatures occurred principally during the initial tissue dissociation and cell preparation and not the cell sorting. The cell preparation-induced activational phenotype could be minimized by inclusion of transcriptional and translational inhibitors or non-enzymatic dissociation conducted entirely at low temperatures. These data demonstrate that a variety of microglial isolation approaches can be used, depending on experimental needs, and that inhibitor cocktails are effective at reducing cell preparation artifacts.

## Visual Abstract

**Figure:**
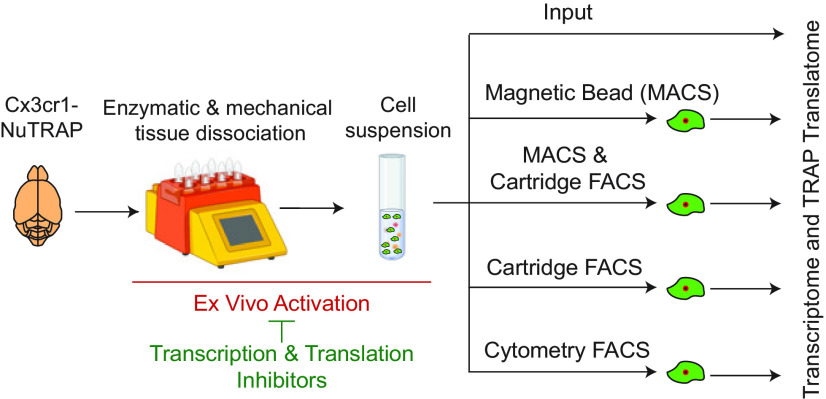

## Significance Statement

Purification of brain microglia from laboratory animal models allows for cell-type specific molecular and biochemical analyses. A long-standing concern of microglial purification is the introduction of artifacts through the isolation process. Comparison of magnetic-activated cell sorting (MACS) and both cytometer-based and cartridge-based fluorescence-activated cell sorting (FACS) show equivalent and sufficient cellular yield/purity and similar levels of *ex vivo* activation which was determined to arise during tissue dissociation. *Ex vivo* microglial activation in cell suspensions generated by a combination of enzymatic and mechanical dissociation procedures was prevented by supplementation with transcription/translation inhibitors during cell preparation. Alternatively, non-enzymatic dissociation conducted at low temperatures prevented such *ex vivo* activational signatures. Cell preparation and sort methods can be further optimized depending on experimental needs.

## Introduction

Microglia, the brain’s resident macrophages, have come to the forefront of neuroimmunology research (Prinz et al., 2019). They serve as surveyors of the CNS and exhibit behavior derived from their embryonic precursors, myeloid cells (Cuadros and Navascués, 1998; Rock et al., 2004), with roles in neurodevelopment, sex differences, as well as in health and neurodegenerative diseases (Salter and Stevens, 2017; Butovsky and Weiner, 2018; Han et al., 2021). Microglial activity is governed by local microenvironments and through communication with neighboring cells. Under stress, microglial cells transition to an activated phenotype, classically defined by morphologic transformation from ramified to amoeboid, release of proinflammatory cytokines, and a shift in global gene expression (Rock et al., 2004; Sierra et al., 2013; Avignone et al., 2015). With the advent of single-cell transcriptomic sequencing, the field has undergone a taxonomy reclassification (Dubbelaar et al., 2018; Provenzano et al., 2021). Current evidence suggests microglia exist within a phenotypic gradient, and the transition away from a quiescent state is no longer viewed as binary “on” or “off.” Thus, the use of microglial gene expression profiles to infer functional status has bolstered the use of transcriptomic profiling as a powerful technique for microglial classification. To delineate the full heterogeneity of microglial population with aging and disease using single-cell techniques, it is necessary to minimize activational effects of cell preparation and isolation.

Traditionally, molecular analyses from specific CNS cell types required the liberation of cells from their native environment and use of fluorescence-activated cell sorting (FACS) or magnetic-activated cell sorting (MACS) labeling techniques before RNA extraction (Cahoy et al., 2008). Cell dissociation primarily consists of enzymatic and/or mechanical dissociation with quality checks for cell viability, debris removal, and absence of cell aggregation (Reichard and Asosingh, 2019). Creation of a single-cell suspension from brain tissue and isolation of microglia is harsh and may alter the phenotypic state of microglia *ex vivo* (Wu et al., 2017; Haimon et al., 2018). *Ex vivo* microglial activation has the potential of introducing confounds that may mask endogenously induced activation, such as in a pathologic state. To avoid cell-isolation confounds, microglial-specific cre-lines (Cx3cr1-cre/ERT2) have been combined with various floxed ribosomal tagging models: (1) ribosome tagging (RiboTag; Haimon et al., 2018); (2) translating ribosome affinity purification (TRAP; Ayata et al., 2018); and (3) nuclear tagging and TRAP (NuTRAP; Chucair-Elliott et al., 2020), allowing the immunoprecipitation (IP) of tagged polysomes to isolate microglial-specific translatomes without the need for cell isolation. Although transgenic ribosome IP approaches overcome many of the potential confounds of *ex vivo* activation, experimental endpoints such as proteomics and single-cell heterogeneity still require cell isolation of intact microglial cells. Additionally, animal availability, complex breeding strategies, and cost will continue to be deterrents for many investigators to using transgenic microglial labeling. While single-cell sequencing allows for broad and potentially unbiased analysis of various cell types, it too is predicated on the creation of a single-cell suspension. Thus, an understanding of the effects of cell preparation and isolation methods on *ex vivo* activation while maintaining highly pure microglial enrichment is needed. The advent of RiboTag approaches allows generation of a reference microglial signature to which sorted microglial profiles can be compared. The goals of this study were to compare purity and yield of isolated microglia and assess the relative level of *ex vivo* activation by comparing Cx3cr1-TRAP-isolated RNA to various sorting techniques used for microglia: MACS (Nikodemova and Watters, 2012; Holt and Olsen, 2016), cytometer-based FACS (Hickman et al., 2013), and newly available low-pressure cartridge-based FACS (Roberts et al., 2021) using the TRAP-enrichment profile as a baseline purified microglial translatome for *ex vivo* activation.

We compared artifacts induced through three cell sorting techniques via transcriptomic profiling of bulk tissue, sorted cells, and immunoprecipitated translatomes and found similar performance with *ex vivo* activational signatures principally occurring during the enzymatic digestion and mechanical dissociation during initial cell preparation. Inclusion of transcriptional and translational inhibitors during the cell preparation step prevented most of these artifacts. Additionally, non-enzymatic tissue dissociation conducted entirely at 4°C was successful at preventing the *ex vivo* activational signature. These studies provide critical insight into the sensitivity of microglia and guidance on experimental design to minimize *ex vivo* confounds of microglial isolation.

## Materials and Methods

### Animals

All animal procedures were approved by the Institutional Animal Care and Use Committee at the Oklahoma Medical Research Foundation (OMRF). Parent mice were purchased from The Jackson Laboratory and bred and housed under SPF conditions in a HEPA barrier environment on a 14/10 h light/dark cycle (lights on at 6 A.M.). Cx3cr1-cre/ERT2^+/+^ males (stock #020940; Yona et al., 2013) were mated with NuTRAP^flox/flox^ females (stock #029899; Roh et al., 2017) to generate the desired progeny, Cx3cr1-cre/ERT2^+/wt^; NuTRAP^flox/wt^ (Cx3cr1-NuTRAP). In the Cx3cr1-NuTRAP mice, following cre recombination in *Cx3cr1*^+^ cells, deletion of the floxed stop cassette causes activation of the NuTRAP allele, labeling microglial ribosomes with eGFP and nuclei with biotin and mCherry. For flow cytometry experiments, cre-negative controls (Cx3cr1-cre/ERT2^wt/wt^ NuTRAP^flox/wt^) were used to establish eGFP gating. DNA was extracted from mouse ear punch samples for genotyping. Mice were approximately three to eight months old at the time of performing experiments. Within each experimental group, both male and female mice were used. The sex of each experimental sample is denoted in Extended Data Figures 2-1, 4-1, 6-1, 7-1. Mice were euthanized by cervical dislocation followed by rapid decapitation. Genotyping primers were obtained from Integrated DNA Technologies and used for PCR detection of (1) generic cre (Jax protocol 22392; primers: oIMR1084, oIMR1085, oIMR7338, oIMR7339); (2) Cx3cr1-cre/ERT2 (Jax protocol 27232; primers: 20669, 21058, 21059); (3) mCherry (Jax protocol 24693; primers: 9703, 9704, oIMR8744, oIMR8745); and (4) NuTRAP floxed allele (Jax protocol 21509; primers: 21306, 24493, 32625, 32626) according to protocols published by The Jackson Laboratory, as previously described (Chucair-Elliott et al., 2020).

### Tamoxifen (Tam) induction of cre recombinase

At approximately three months, mice received a daily intraperitoneal injection of Tam solubilized in 100% sunflower seed oil by sonication (100 mg/kg body weight, 20 mg/ml stock solution, #T5648; MilliporeSigma) for five consecutive days, as previously (Chucair-Elliott et al., 2019). Based on an average weight of 20 g per mouse, each daily injection of Tam consisted of 100 μl of 20 mg/ml stock solution. Adjustments were made for mice that significantly deviated from the average weight. Tissues were harvested from five- to eight-month mice, allowing at least two months following the last Tam injection and turnover of CX3CR1^+^ circulating cells.

### Flow cytometry from blood

Blood was collected from the facial vein of Cx3cr1-NuTRAP (3 and 60 d after Tam induction) and cre-negative (WT) controls (*n* = 3–10/group) using a 5 mm sterile Goldenrod animal lancet (MEDIpoint) and mixed with 10 μl 0.5 m EDTA, pH 8.0 to avoid coagulation (Chucair-Elliott et al., 2020). Ten volumes of room temperature 1× Red Blood Cell Lysis solution (#130-094-183, Miltenyi Biotec) were added to one volume of collected blood, vortexed for 5 s, and then incubated in the dark at room temperature for 10 min. Cells were pelleted at 300 × *g* for 10 min at room temperature and then the supernatant was decanted. The cells were resuspended in 1 ml 0.5% BSA (#130-091-376, Miltenyi Biotec) in 1× D-PBS containing calcium, magnesium, glucose, and pyruvate (#14287-072, Thermo Fisher Scientific) and then washed by centrifugation at 300 × *g* for 10 min at room temperature. The cell pellet was resuspended in 200 μl 0.5% BSA in 1× D-PBS and split into 50 μl aliquots for antibody staining. Cell aliquots were then stained with the following: (1) 1 μl of CD11b-APC (M1/70.15.11.5, #130-113-793, Miltenyi Biotec); (2) 1 μl of CD45-VioBlue (REA737, #130-110-802, Miltenyi Biotec); (3) 1 μl of CD11b-APC and 1 μl of CD45-VioBlue; or (4) left unstained as a control. Stained cells were gently mixed and then incubated for 10 min in the dark at 4°C. Cells were then washed with 1 ml of 0.5% BSA in 1× D-PBS and then pelleted at 300 × *g* for 10 min at room temperature. The cell pellet was resuspended in 250 μl of 0.5% BSA in 1× D-PBS before flow cytometric analysis on the MACSQuant Analyzer 10 (#130-096-343, Miltenyi Biotec).

### Preparation of single-cell suspension from mouse brain using a combination of enzymatic and mechanical dissociation

Halves of Cx3cr1-NuTRAP mouse brains were rinsed in ice-cold D-PBS containing calcium, magnesium, glucose, and pyruvate (#14287-072, Thermo Fisher Scientific), sliced into four sagittal sections on a chilled, metal block and placed into ice-cold gentleMACS C-tubes (#130-093-237, Miltenyi Biotec), containing 1950 μl of papain-based, Enzyme Mix 1. For each reaction, Enzyme Mix 1 was created by combining 50 μl of Enzyme P with 1900 μl of buffer Z, while Enzyme Mix 2 was created by combining 10 μl of Enzyme A with 20 μl of buffer Y per reaction, with all reagents included in the Adult Brain Dissociation kit (#130-107-677, Miltenyi Biotec). Each sample then had 30 μl of Enzyme Mix 2 added before being mechanically dissociated for 30 min at 37°C on the gentleMACS Octo Dissociator with Heaters (#130-096-427, Miltenyi Biotec) using the 37C_ABDK_01 program. Following enzymatic and mechanical dissociation, the C-tubes were quickly spun in a chilled (4°C) Allegra-30R centrifuge (#B08708, Beckman Coulter) with an SX4400 swinging bucket rotor to collect the samples in the bottom of the tubes. Next, samples were resuspended and passed through a prewet 70 μm MACS SmartStrainer (#130-110-916, Miltenyi Biotec) and collected in a 50-ml conical tube (#21008-178, VWR International). The C-tubes were washed with 10 ml of ice-cold D-PBS and the washed volume was passed through the 70 μm MACS SmartStrainer. The cells were then pelleted by centrifugation at 300 × *g* for 10 min at 4°C. Following centrifugation, the supernatant was aspirated and debris was removed using the Debris Removal solution (#130-109-398, Miltenyi Biotec) provided in the Adult Brain Dissociation kit (#130-107-677, Miltenyi Biotec). Briefly, cells were resuspended in 3.1 ml of ice-cold D-PBS and passed to a 15-ml conical tube (#21008-214, VWR International) and 900 μl of cold Debris Removal solution was mixed into the cell suspensions. Next, 4 ml of D-PBS was gently overlaid on top of the cell suspension, ensuring the layers did not mix. Centrifugation at 3000 × *g* for 10 min at 4°C separated the suspension into three phases, of which the top two phases were aspirated. The cell pellet was gently resuspended in 15 ml of ice-cold D-PBS before centrifugation at 1000 × *g* for 10 min at 4°C. After aspirating the supernatant completely, the cells were resuspended in 1 ml 0.5% BSA (#130-091-376, Miltenyi Biotec) in D-PBS and filtered through a 35-μm filter (#352235, Fisher Scientific). A 100 μl aliquot of cells was retained as “Cell-Input” for flow cytometric and RNA sequencing (RNA-Seq) analyses.

### Preparation of single-cell suspension from mouse brain using mechanical dissociation

As a comparator to the enzymatic and mechanical dissociation, which requires a 30-min incubation at elevated temperatures (37°C), single-cell suspensions were also created using mechanical dissociation alone with all steps conducted at 4°C or on ice. The protocol for this cell preparation was modified from a previously published study (Hammond et al., 2019). Briefly, halves of Cx3cr1-NuTRAP mouse brains were rinsed in ice-cold 1× HBSS with no calcium, no magnesium, and no phenol red (diluted 1:10 from #14185-052, ThermoFisher), sliced into four sagittal sections and placed into a 7-ml Kimble Dounce homogenizer (#D9063, MilliporeSigma) containing 5-ml prechilled 1× HBSS. The brain tissue was homogenized using 15 strokes of the loose and tight pestles while simultaneously rotating the pestle and then passed through a prewet 70-μm cell strainer (#130-095-823, Miltenyi Biotec) placed onto a prechilled 15-ml conical tube. The glass homogenizer was washed twice with 5 ml of 1× HBSS and the washed volume was transferred to the 15-ml conical tube containing the brain homogenate. Next, cells were pelleted at 300 × *g* for 5 min at 4°C. Following centrifugation, the supernatant was aspirated and the cells were resuspended in 10 ml 40% ice-cold Percoll (#17-0891-02, MilliporeSigma) in 1× HBSS (4 ml Percoll, 1 ml 10× HBSS, 5 ml RNase-free water). The cell mixture was spun at 500 × *g* for 30 min at 4°C using full acceleration and braking. The myelin and Percoll were carefully aspirated, leaving the microglia-containing cell pellet undisturbed. The cell pellet was then washed in 10 ml of ice-cold HBSS and spun again at 300 × *g* for 5 min at 4°C. After aspirating the supernatant completely, the cells were resuspended in 1 ml 0.5% BSA (#130-091-376, Miltenyi Biotec) in D-PBS and filtered through a 35-μm filter (#352235, Fisher Scientific). An aliquot of 200-μl cells was retained as “Dounce-Input” for flow cytometry. The remaining cells were pelleted at 300 × *g* for 10 min at 4°C to process for TRAP and RNA-Seq library preparation.

### Cell counting from brain tissue

Filtered cells were diluted 1:10 in 0.5% BSA (#130-091-376, Miltenyi Biotec) in D-PBS before cell counting on a MACSQuant Analyzer 10 (#130-096-343, Miltenyi Biotec). A total of 50 μl of diluted cells were analyzed to determine absolute cell count. Cells were gated on FSC-A/SSC-A to determine cell count and FSC-A/FSC-H to determine singlet count. Absolute cell counts were used to determine antibody staining ratios.

### CD11b magnetic labeling and separation from brain tissue

Cells from a single hemisphere of a Cx3cr1-NuTRAP mouse brain were pelleted at 300 × *g* for 10 min at 4°C and resuspended in 90 μl of 0.5% BSA in D-PBS with 10 μl of CD11b (Microglia) MicroBeads (#130-093-636, Miltenyi Biotec) per 10^7^ total cells. After gently pipetting to mix, cells were incubated for 15 min at 2–8°C protected from light. Cells were washed with 1 ml of 0.5% BSA in D-PBS and pelleted at 300 × *g* for 10 min at 4°C. The cell pellet was resuspended in 500 μl of 0.5% BSA in D-PBS. After priming the autoMACS Pro Separator (#130-092-545, Miltenyi Biotec), sample and collection tubes were placed in a cold MACS Chill 5 Rack (#130-092-951, Miltenyi Biotec) with both positive and negative fractions being collected. The double-positive selection (Posseld) program (i.e., positive fraction cells are then run over a second magnetic column for higher purity) was used to elute highly pure CD11b^+^ cells in 500 μl of autoMACS Running buffer (#130-091-221, Miltenyi Biotec). Following separation, the positive fraction was reserved for further applications and analysis.

### Antibody labeling for FACS from brain tissue

Cell suspensions from a single hemisphere of a Cx3cr1-NuTRAP mouse brain were pelleted at 300 × *g* for 10 min at 4°C and resuspended in 96 μl of 0.5% BSA in D-PBS, 2 μL of CD11b-APC antibody (M1/70.15.11.5, #130-113-793, Miltenyi Biotec), and 2 μl of CD45-VioBlue antibody (REA737, #130-110-802, Miltenyi Biotec). After mixing well, cells were incubated for 10 min in the refrigerator (2–8°C) protected from light. Cells were washed with 1 ml of 0.5% BSA in D-PBS and pelleted at 300 × *g* for 10 min. Cell suspensions from half brains were processed in parallel for cartridge-based FACS (MACSQuant Tyto) and cytometer-based FACS (FACSAria).

### Cartridge-based FACS (MACSQuant Tyto) from brain tissue

Stained cell pellets from a single hemisphere of a Cx3cr1-NuTRAP mouse brain were resuspended in 10 ml of 0.5% BSA in D-PBS. A MACSQuant Tyto Cartridge (#130-106-088, Miltenyi Biotec) was primed using 1 ml of 0.5% BSA in D-PBS. The cell suspension was then filtered through 20-μm Pre-Separation Filters (#130-101-812, Miltenyi Biotec). An aliquot of 500 μL of filtered cell suspension was saved as the Tyto-Input fraction for analysis. The remaining cell suspension was then loaded into the input chamber of a MACSQuant Tyto cartridge. After loading labeled cells into the input chamber, the cartridge was scanned into the MACSQuant Tyto Cell Sorter system (#130-103-931, Miltenyi Biotec) and sorting parameters were selected. The MACSQuant Tyto cartridge is a sterile, closed, single-use system that relies on accurate activation of the sort valve to pass cells of interest (in this case microglia) into the positive sort chamber. Cell speed (or time-of-flight) was determined by the time it took a cell to pass between two adjacent lasers. In this study, the V1 filter (450/50 nm) of the violet (405 nm) laser was used as a cell trigger, the first PMT channel used to measure cell speed, to detect CD45-VioBlue positive cells at a threshold signal of 20. The B1 filter (525/550) of the blue (488 nm) laser was used as the cell speed channel to detect eGFP+ cells at a signal threshold of 4. A blue (488 nm) laser with B1 (525/50 nm) and B2 (585/40 nm) filter combinations was used to gate on eGFP^+^ cells without auto-fluorescence interference. Subsequent gating based on CD11b-APC fluorescence used a red (638 nm) laser and R1 (655–730 nm) filter. The gating strategy was set to CD11b^+^CD45^+^eGFP^+^ for isolation of microglia (Extended Data Fig. 1-5). After completion of the sort, the cells from the positive fraction chamber were collected in 0.5% BSA in D-PBS. The positive fraction chamber was washed twice using 450 μl of 0.5% BSA in D-PBS per wash and combined with the initial positive fraction collection. After sorting was completed, an aliquot of (10%) of the positive fraction was kept for analysis on the MACSQuant Analyzer 10 Flow Cytometer.

### Cytometer-based FACS (FACSAria) from brain tissue

Following staining, cells from a single hemisphere of a Cx3cr1-NuTRAP mouse brain were pelleted and then resuspended in 2 ml of 0.5% BSA in D-PBS for cytometer-based sorting (FACSAria IIIu cell sorter, BD Biosciences). An aliquot of 200-μl stained cells was saved as FACS-Input for analysis. A violet (405 nm) laser was used to gate CD45-VioBlue-positive cells using a 450/40-nm filter. A blue (488 nm) laser with 530/30 nm and yellow-green (561 nm) laser with 610/20 nm filter combinations was used to gate on eGFP^+^ cells without auto-fluorescence interference. A red (640 nm) laser was used to detect CD11b-APC fluorescence with a 660/20-nm filter. The gating strategy was set to CD11b^+^CD45^+^eGFP^+^ for isolation of microglia (Extended Data Fig. 1-6). Cells were sorted into 500 μl of 0.5% BSA in D-PBS. After sorting was completed, an aliquot (10%) of the positive fraction was analyzed on the MACSQuant Analyzer 10 Flow Cytometer.

### Addition of inhibitors

Transcription and translation inhibitors were included during cell preparation, as previously described (Marsh et al., 2020) with some minor modifications. Actinomycin D (#A1410, MilliporeSigma) was reconstituted in DMSO to a concentration of 5 mg/ml before being aliquoted and stored at −20°C protected from light. Triptolide (#T3652, MilliporeSigma) was reconstituted in DMSO to a concentration of 10 mm before being aliquoted and stored at −20°C protected from light. Anisomycin (#A9789, MilliporeSigma) was reconstituted in DMSO to a concentration of 10 mg/ml before being aliquoted and stored at 4°C protected from light. For the samples to be treated with inhibitors, 2 μl each of actinomycin D, triptolide, and anisomycin stocks were added to the initial Enzyme Mix 1 before dissociation for a final concentration of 5 μg/ml, 10 μm, and 10 μg/ml, respectively. The transcription and translation inhibitors were only included during the enzymatic and mechanical dissociation step for 30 min at 37°C on the gentleMACS Octo Dissociator with Heaters (#130-096-427, Miltenyi Biotec) using the 37C_ABDK_01 program. The transcription and translation inhibitors were not present in the dissection or sorting buffers, as the cell preparation was kept at 4°C for all other steps.

### Flow cytometry analysis from brain tissue

For analysis of cell sorting, aliquots of input and positive fractions from each of the sort methods (autoMACS, autoMACS to MACSQuant Tyto, MACSQuant Tyto, FACSAria) were taken for analysis on the MACSQuant Analyzer 10 Flow Cytometer. autoMACS input and positive fractions were stained with CD11b-APC (M1/70, #130-113-793, Miltenyi Biotec) and CD45-VioBlue (REA737, #130-110-802, Miltenyi Biotec) after completion of the sort, according to manufacturer’s instructions. autoMACS to MACSQuant Tyto, MACSQuant Tyto, and FACSAria input and positive fractions were stained before cell sorting. Following staining, cells were resuspended in 500 μl of 0.5% BSA/D-PBS and run on the MACSQuant Analyzer 10 Flow Cytometer. Postsort purity was assessed by (1) percent eGFP^+^ singlets and (2) percent CD11b^+^CD45^+^ singlets (Extended Data Fig. 1-7) using MACSQuantify v2.13.0 software.

To test the effect of cell preparation method on the relative abundance of cell types, aliquots of cells were stained with the following: (1) microglial [CD11b-APC (M1/70, #130-113-793, Miltenyi Biotec)/CD45-VioBlue (REA737, #130-110-802, Miltenyi Biotec)]; (2) neuronal [CD24-VioBlue (REA743, #130-110-831, Miltenyi Biotec)]; (3) astrocytic [ACSA2-APC (REA969, #130-116-245, Miltenyi Biotec)]; or (4) Oligodendrocytic [O4-APC (REA576, #130-119-982, Miltenyi Biotec)] fluorophore-conjugated antibodies, according to manufacturer’s instructions. Cells were washed and resuspended in 500 μl and run on the MACSQuant Analyzer 10 Flow Cytometer. Relative cell proportions with and without transcription/translation inhibitors were assessed (Extended Data Fig. 6-3) using MACSQuantify v2.13.0 software.

To test the effect of cell preparation method on the viability of single-cell suspensions, aliquots of cells were stained with Viobility 405/520 Fixable Dye (#130-110-206, Miltenyi Biotec), according to manufacturer’s instructions. Briefly, 100 μl of cells in 1× D-PBS were incubated with 1 μl of Viobility 405/520 Fixable Dye for 15 min in the dark at room temperature. After washing with 1 ml of 0.5% BSA/D-PBS, the cells were resuspended in 250 μl of 0.5% BSA/D-PBS and run on the MACSQuant Analyzer 10 Flow Cytometer. The percent viable singlets and eGFP^+^ singlets were assessed (Extended Data Fig. 6-4) using MACSQuantify v2.13.0 software.

10.1523/ENEURO.0348-21.2022.f6-4 Extended Data Figure 6-4 Comparison of cell viability between different cell preparation methods. Following creation of a single-cell suspension, cell viability was assessed by flow cytometry. ***A***, ***B***, Gating strategy to determine viability of all single cells and eGFP^+^ cells. ***C***, There was no difference in overall cell viability between the different cell preparation methods. ***D***, Mechanical dissociation carried out entirely at 4°C (“Dounce”) had slightly lower viability among eGFP^+^ singlets than the enzymatic and mechanical dissociation [“Cell (+/–) Inhib”]. Inclusion of transcription and translation inhibitors did not alter cell viability (one-way ANOVA, Tukey’s *post hoc* test, **p < 0.01). Boxplots represent median, Q1, Q3, minimum, and maximum of each dataset. Download Figure 6-4, EPS file.

### TRAP and RNA extraction

For TRAP from whole tissue, a hemisected half-brain was minced into small pieces and homogenized in 1.5 ml ice-cold complete homogenization buffer: 50 mm Tris, pH 7.4; 12 mm MgCl_2_; 100 mm KCl; 1% NP-40; 1 mg/ml sodium heparin; 1 mm DTT; 100 μg/ml cycloheximide (#C4859-1ML, MilliporeSigma); 200 units/ml RNaseOUT Recombinant Ribonuclease Inhibitor (#10777019; Thermo Fisher Scientific); 0.5 mm Spermidine (#S2626, MilliporeSigma), 1× complete EDTA-free Protease Inhibitor Cocktail (#11836170001; MilliporeSigma) with a glass Dounce tissue grinder set (#D8938; MilliporeSigma), as previously described (Chucair-Elliott et al., 2020). For TRAP from cells, a single hemisphere of a Cx3cr1-NuTRAP mouse brain was used to generate a cell suspension before sorting. After pelleting cells at 1000 × *g* for 10 min at 4°C, cells were resuspended in 400 μl of ice-cold complete homogenization buffer, transferred to a glass Dounce tissue grinder set, and homogenized 15 times with pestle A. Volume was brought up to 1.5 ml with complete homogenization buffer. Homogenates (from tissue or cells) were transferred to 2 ml round-bottom tubes and centrifuged at 12,000 × *g* for 10 min at 4°C. After centrifugation, 100 μl of the supernatant was saved as TRAP “Input.” The remaining supernatant was transferred to a 2-ml round-bottom tube and incubated with 5 μg/μl of anti-GFP antibody (ab290; Abcam) at 4°C with end-over-end rotation for 1 h. Dynabeads Protein G for IP (#10003D; Thermo Fisher Scientific) were washed three times in 1-ml ice-cold low-salt wash buffer (50 mm Tris, pH 7.5; 12 mm MgCl_2_; 100 mm KCl; 1% NP-40; 100 μg/ml cycloheximide; 1 mm DTT). After removal of the last wash, the homogenate/antibody mixture was transferred to the 2-ml round-bottom tube containing the washed Protein-G Dynabeads and incubated at 4°C with end-over-end rotation overnight. Magnetic beads were collected using a DynaMag-2 magnet and the unbound-ribosomes and associated RNA discarded. Beads and GFP-bound polysomes were then washed three times with 0.5 ml of high-salt wash buffer (50 mm Tris, pH 7.5; 12 mm MgCl_2_; 300 mm KCl; 1% NP-40; 100 μg/ml cycloheximide; 2 mm DTT). Following the last wash, 350 μl of buffer RLT (QIAGEN) supplemented with 3.5 μl 2-β mercaptoethanol (#444203, MilliporeSigma) was added directly to the beads and incubated with mixing on a ThermoMixer (Eppendorf) for 10 min at room temperature. The beads were magnetically separated and the supernatant containing the target bead-bound polysomes and associated RNA was transferred to a new tube. A total of 350 μl of 100% ethanol was added to the tube (“TRAP” fraction: enriched in transcriptome associated to eGFP-tagged ribosomes) and then loaded onto a RNeasy MinElute column (QIAGEN). RNA was isolated using RNeasy Mini kit (#74104, QIAGEN), according to manufacturer’s instructions. RNA was quantified with a Nanodrop One^c^ spectrophotometer (#ND-ONEC-W, Thermo Fisher Scientific) and its quality assessed by HSRNA ScreenTape (#5067-5579, Agilent Technologies) with a 4150 Tapestation analyzer (#G2992AA, Agilent Technologies).

### Library construction and RNA-Seq

Directional RNA-Seq libraries were made according to the manufacture’s protocol from 2 to 100 ng RNA. TRAP input and positive fraction RNA from all samples were used to create individual RNA-Seq libraries (no pooling of samples was performed). Briefly, poly-adenylated RNA was captured using NEBNext Poly(A) mRNA Magnetic Isolation Module (#NEBE7490L; New England Biolabs, Ipswich, MA). Following mRNA capture, mRNA was eluted from the oligo-dT beads and fragmented by incubating with the First Strand Synthesis Reaction buffer and Random Primer Mix (2×) from the NEBNext Ultra II Directional Library Prep Kit for Illumina (#NEBE7760L; New England Biolabs) for 15 min at 94°C. First and second strand cDNA synthesis were performed sequentially, as instructed by the manufacturer’s guidelines. After purification of double-stranded cDNA with 1.8× SPRISelect Beads (#B23318, Beckman Coulter), purified cDNA was eluted in 50 μl 0.1× TE buffer and subjected to end prep. The NEBNext Adaptor was diluted 1:100 in Adaptor Dilution buffer (provided) before ligating the adaptor to the cDNA. After purifying the ligation reaction with 0.9× SPRISelect Beads (#B23318, Beckman Coulter), cDNA was eluted in 15 μl of 0.1× TE (provided). Next, cDNA libraries were amplified with 16 cycles of PCR using the NEBNext Ultra II Q5 Master Mix (provided) and unique index primer mixes from NEBNext Multiplex Oligos for Illumina Library (#E6609L, New England Biolabs). Libraries were purified with 0.9× SPRISelect Beads (#B23318, Beckman Coulter) and then sized with HSD1000 ScreenTapes (#5067-5584; Agilent Technologies). Libraries had an average peak size of 316 bp. Libraries were quantified by Qubit 1× dsDNA HS Assay kit (#Q33230, Thermo Fisher Scientific). The libraries for each sample were pooled at 4 nm concentration and sequenced using an Illumina NovaSeq 6000 system (SP PE50bp, S4 PE150). The entirety of the sequencing data are available for download in FASTQ format from NCBI Gene Expression Omnibus (to be included on acceptance).

### RNA-Seq data analysis

Following sequencing, reads were trimmed and aligned before differential expression statistics and correlation analyses in Strand NGS software package (v4.0; Strand Life Sciences). Reads were aligned against the full mm10 genome build (2014.11.26). Alignment and filtering criteria included the following: adapter trimming, fixed 2-bp trim from 5′ and 2 bp from 3′ ends, a maximum number of one novel splice allowed per read, a minimum of 90% identity with the reference sequence, a maximum 5% gap, and trimming of 3′ end with Q < 30. Alignment was performed directionally with Read 1 aligned in reverse and Read 2 in forward orientation. All duplicate reads were then removed. Normalization was performed with the DESeq2 algorithm. Transcripts with an average read count value >5 in at least 100% of the samples in at least one group were considered expressed at a level sufficient for quantitation per tissue and those transcripts below this level were considered not detected/not expressed and excluded, as these low levels of reads are close to background and are highly variable. For statistical analysis of differential expression, a one-way ANOVA or two-way ANOVA with the factors of TRAP fraction and treatment and a Benjamini–Hochberg multiple testing correction (BHMTC) followed by Student–Newman–Keuls *post hoc* test were used (FDR < 0.1). For those transcripts meeting this statistical criterion, a fold change >|2| cutoff was used to eliminate those genes which were statistically significant but unlikely to be biologically significant and orthogonally confirmable because of their very small magnitude of change. Visualizations of hierarchical clustering and principal component analyses (PCAs) were performed in Strand NGS (version 4.0). Cell type specific marker gene lists were generated from the re-analysis published previously (McKenzie et al., 2018) of immunopurified and high throughput single-cell data from mice. Overrepresentation analysis (ORA) was conducted using WEB-based Gene SeT AnaLysis Toolkit (WebGestalt; www.webgestalt.org; Liao et al., 2019). Top overrepresented biological processes were selected from gene ontology functional database with no redundant option selected (hypergeometric test, BHMTC, FDR < 0.05) and background reference gene list of all expressed genes (raw count > 5 in all samples from at least one group). Top overrepresented transcription factor targets were selected from network functional database with all expressed genes as the reference gene list (hypergeometric test, BHMTC, FDR < 0.05). Heatmaps of overrepresented biological processes and gene lists were created using Morpheus (https://software.broadinstitute.org/morpheus). Upset plots were created using UpSetR v1.4.0 package in RStudio v1.4.1106 with R v4.0.5. Previously published microglial *ex vivo* activational lists were compared (Ayata et al., 2018; Haimon et al., 2018; Marsh et al., 2020) and genes included in at least two of the three previous studies were considered “*ex vivo* activational transcripts.” Gene set enrichment analysis (GSEA) was run using GSEA v4.1.0 (Mootha et al., 2003; Subramanian et al., 2005).

### Immunochemistry and imaging

Brain samples were fixed for 4 h in 4% PFA, cryoprotected by sequential incubations in PBS containing 15% and 30% sucrose, and then frozen in Optimal Cutting Temperature medium (#4583, Tissue-Tek). Twelve μm-thick sagittal sections were cryotome-cut (Cryostar NX70, Thermo Fisher Scientific). Tissue sections were rinsed with PBS containing 1% Triton X-100, blocked for 1 h in PBS containing 10% normal donkey serum, and processed for fluorescence immunostaining and downstream analysis. The primary antibodies included rabbit anti-GFP (#ab290, 1:100, Abcam), rat anti-CD11b (#C227, Clone M1/70, 1:100, Leinco Technologies), and rat anti-CD45 (#550539, Clone 30-F111, 1:100, BD Biosciences). Sequential imaging was performed on an Olympus FluoView confocal laser-scanning microscope (FV1200; Olympus) at the Dean McGee Eye Institute imaging core facility at OUHSC. Microscope and FLUOVIEW FV1000 version 1.2.6.0 software (Olympus) settings were identical for samples using the same staining-antibody combination and at same magnification. The experimental format files were oib. The Z-stack generated was achieved at 1.26-μm step size with a total of eight optical slices at 20× magnification (2× zoom).

## Results

**Figure 1.**
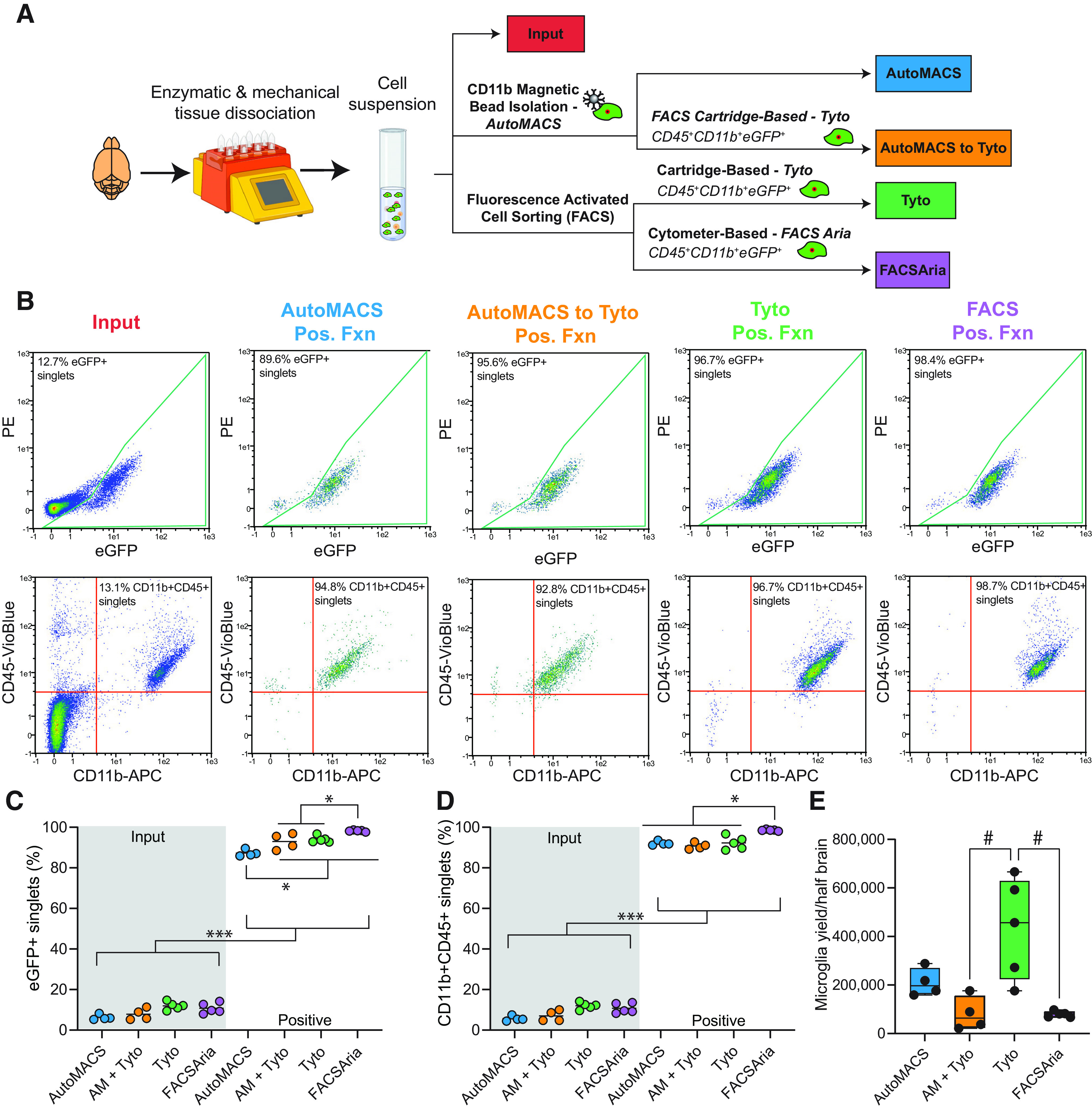
Comparison of purity and yield among microglial cell isolation techniques. ***A***, Schematic of the experimental design. Cx3cr1-NuTRAP brains were enzymatically and mechanically dissociated to create a single-cell suspension. Different microglial sorting techniques were compared with Cell-Input for purity, yield, and transcriptomic signatures. ***B***, Representative flow cytometry plots of immunostained single-cell suspensions from input and after each of the sorting strategies shows a distinct population of eGFP^+^ cells (identified as *Cx3cr1*^+^ microglia) and CD11b^+^CD45^+^ cells (identified as microglia per traditional cell surface markers, as shown by IHC in Extended Data Fig. 1-2). Assessment of the loss of eGFP in circulating CD11b^+^CD45^+^ cells 60 d after Tam induction (Extended Data Fig. 1-3) and the relative levels of CD45^mid^ and CD45^high^ cell populations from the CD11b^+^CD45^+^ each sort method and cell input from brain (Extended Data Fig. 1-4) were also performed to further characterize the sort fractions. Representative plots of the gating strategies used for the Tyto sort (Extended Data Fig. 1-5), FACSAria sort (Extended Data Fig. 1-6), and flow cytometry (Extended Data Fig. 1-7) are given as supplements. ***C***, ***D***, All sort positive fractions were enriched for (***C***) eGFP^+^ singlets and (***D***) CD11b^+^CD45^+^ singlets (as compared with input; two-way ANOVA, main effect of TRAP fraction, *p < 0.05, ***p < 0.001; Extended Data Fig. 1-1). When comparing positive fractions, the autoMACS positive fraction had lower % eGFP^+^ singlets as compared with all other sort methods. FACSAria had higher percentage of eGFP^+^ singlets than all other sort methods. FACSAria had higher percentage of CD11b^+^CD45^+^ singlets as compared with all other sort methods positive fractions (two-way ANOVA, Tukey’s *post hoc* test, *p < 0.05). ***E***, Microglial yield was significantly higher in the MACSQuant Tyto positive fraction as compared with the autoMACS to MACSQuant Tyto and FACSAria positive fractions (one-way ANOVA, Tukey’s *post hoc* test, #*p* < 0.01). Created with BioRender.com.

The goal of this study was to compare microglial sorting techniques and determine the relative levels of *ex vivo* activation induced during cell preparation and microglial isolation. A schematic of the experimental design is represented in Figure 1*A*. In the Cx3cr1-NuTRAP mice, following cre recombination in *Cx3cr1*^+^ cells, deletion of the floxed stop cassette causes activation of the NuTRAP allele, labeling microglial ribosomes with eGFP and nuclei with biotin and mCherry. For the first part of the present study, we used eGFP as a sorting criterion and in the evaluation of postsort microglial purity, along with CD11b and CD45 co-expression. Colocalization of eGFP with microglial markers CD11b and CD45 in Cx3cr1-NuTRAP brains was verified by immunohistochemistry (Extended Data Fig. 1-2). Enzymatic and mechanical dissociation of Cx3cr1-NuTRAP brains was performed to generate single-cell suspensions. For each sort method, both male and female samples were used.

10.1523/ENEURO.0348-21.2022.f1-1 Extended Data Figure 1-1 Figure 1 source data. Download Figure 1-1, XLS file.

**Figure 2.**
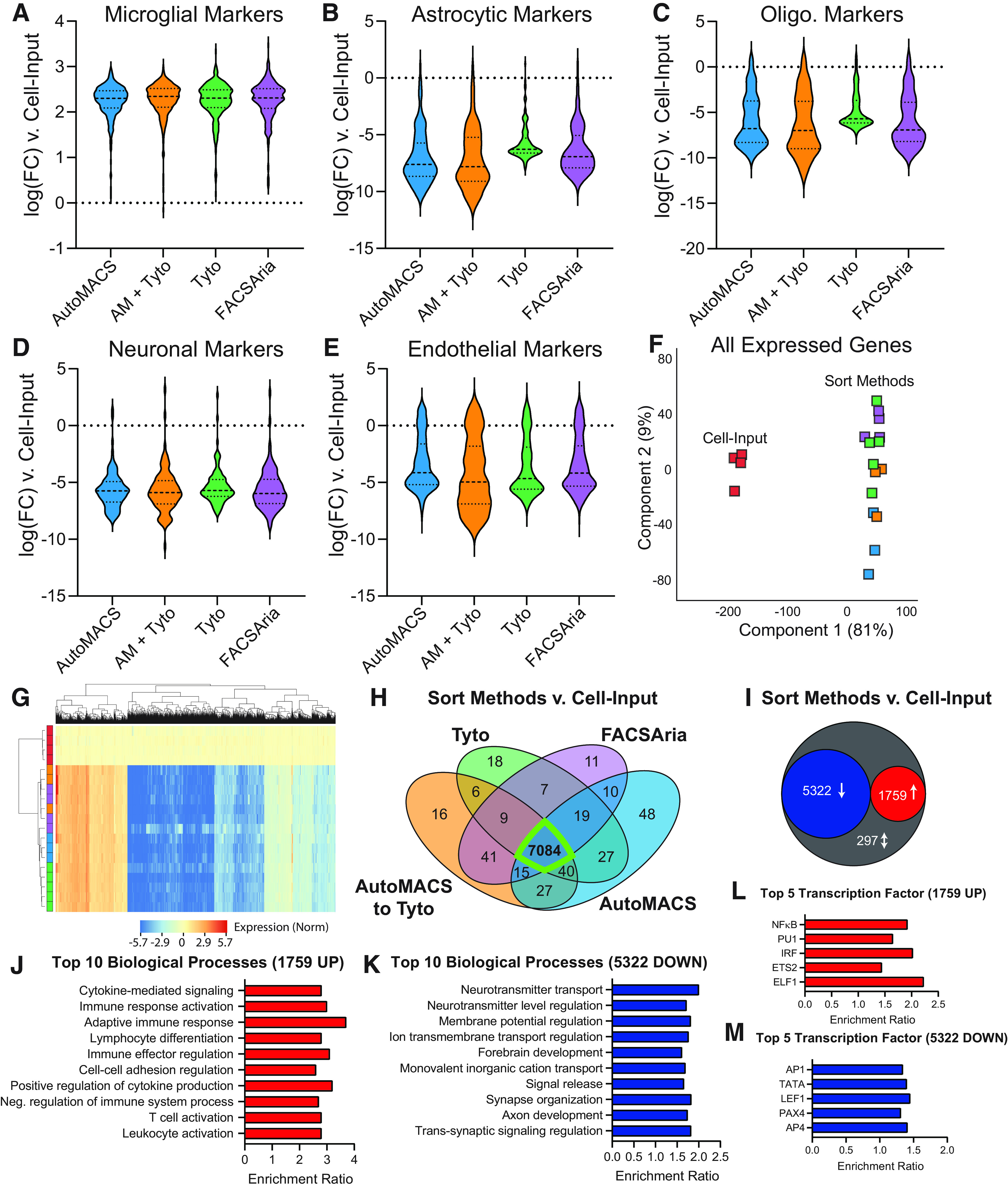
Comparison of transcriptomic profiles of microglia isolated from various cell sorting methods. RNA-Seq libraries were made from each of the groups represented in Figure 1*A* to compare the transcriptomic profiles of microglia isolated via four different cell sorting strategies. Basic sequencing metrics and all source data for Figure 2 are provided in Extended Data Figure 2-1. Gene body coverage plots showed similar coverage across the gene bodies in each group (Extended Data Fig. 2-2). Each of the strategies had similar levels of (***A***) enrichment of microglial transcripts and depletion of (***B***) astrocytic, (***C***) oligodendrocytic, (***D***) neuronal, and (***E***) endothelial transcripts when compared with Cell-Input. ***F***, PCA of all expressed genes shows clear separation of Cell-Input from all other sort methods in the first component with 81% of explained variance. ***G***, Hierarchical clustering of differentially expressed genes (DEGs; one-way ANOVA, BHMTC, SNK FDR < 0.1, |FC|>2) shows separation of Cell-Input and sort methods. Each of the sort methods show very similar patterning of expression across DEGs. ***H***, Comparison of SNK *post hocs* from each of the sort methods versus Cell-Input, showed the majority of enrichments/depletions (i.e., DEGs; 7084/7378 = 96%) were in common between all sort methods. ***I***, There were 5322 DEGs (72%) that were depleted and 1759 DEGs (24%) that were enriched in all sort methods compared with Cell-Input. There were only 297 discordant DEGs (4%) between the different sort methods as compared with Cell-Input. ***J***, Top 10 biological processes overrepresented in the 1759 genes upregulated in all sort methods compared with Cell-Input (Gene Ontology ORA, BHMTC FDR <0.05). ***K***, Top 10 biological processes overrepresented in the 5322 genes downregulated in all sort methods compared with Cell-Input (Gene Ontology ORA, BHMTC FDR <0.05). ***L***, Top five transcription factor targets overrepresented in the 1759 genes upregulated in all sort methods compared with Cell-Input (transcription factor target network ORA, BHMTC FDR < 0.05). ***M***, Top five transcription factor targets overrepresented in the 5322 genes downregulated in all sort methods compared with Cell-Input (transcription factor target network ORA, BHMTC FDR < 0.05).

10.1523/ENEURO.0348-21.2022.f1-2 Extended Data Figure 1-2 Validation of microglial identity of recombined cells in the Cx3cr1-NuTRAP brain. Two months after Tam treatment, brains were harvested from Cx3cr1-NuTRAP and cre-negative NuTRAP^+^ (control) mice for immunohistochemistry (IHC). ***A***, Representative confocal fluorescent microscopy images of sagittal brain sections captured in the cortex show eGFP expression (green signal) in cells that co-expressed CD11b (red signal, ***a***, ***a’***, ***b***, ***b’***) and CD45 (red signal, ***c***, ***c’***, ***d***, ***d’***) in Cx3cr1-NuTRAP brains but not in the cre-negative counterparts (*n* = 2/group). ***B***, Representative confocal fluorescent microscopy images captured in the hippocampus show eGFP expression (green signal) in cells that co-expressed CD11b (***a***, ***a’***) and CD45 (***b***, ***b’***) in Cx3cr1-NuTRAP brains DAPI: nuclear counterstain. Scale bar: 50 μm Download Figure 1-2, TIF file.

**Figure 3.**
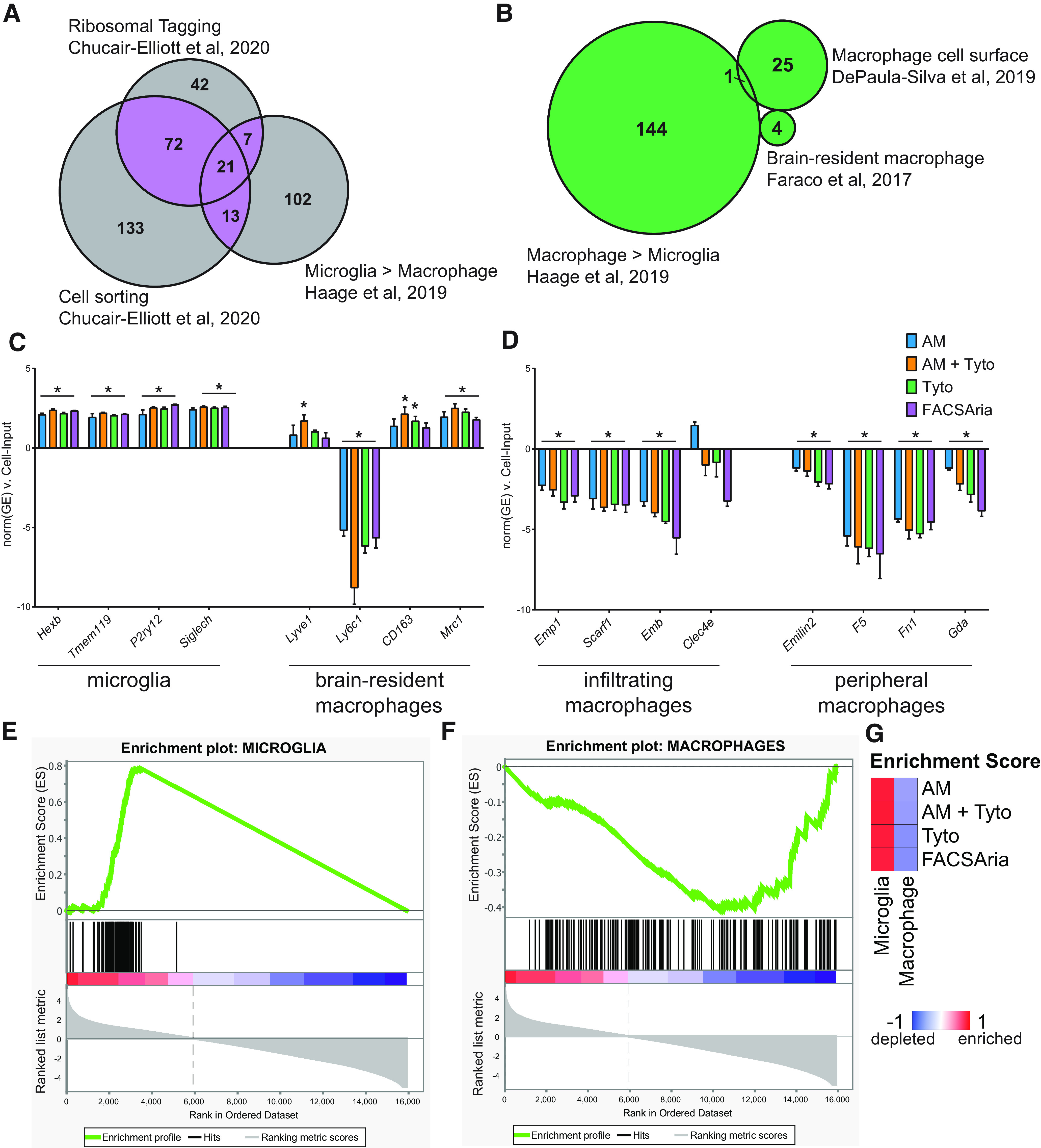
Assessment of expression of non-microglial macrophage markers in sorted cells from Cx3cr1-NuTRAP brains. Gene marker lists and source data for Figure 3 are provided in Extended Data Figure 3-1. ***A***, Previously curated microglial marker gene lists from cell sorting (Haage et al., 2019; Chucair-Elliott et al., 2020) and ribosomal tagging (Chucair-Elliott et al., 2020) approaches were compared for overlap. Microglial marker genes observed in at least two of the studies (113 genes, purple overlap) were used for further analysis (Extended Data Fig. 3-1). ***B***, Previously curated non-microglial macrophage marker gene lists from peripheral monocytes/macrophages (Haage et al., 2019), non-microglia brain resident macrophages (Faraco et al., 2017), and infiltrating macrophages (DePaula-Silva et al., 2019) were compared. The union of all macrophage markers (174 genes, green) was used for further analysis (Extended Data Fig. 3-1). ***C***, RNA-Seq gene expression of brain-resident macrophage marker genes from (1) microglia (*Hexb*, *Tmem119*, *P2ry12*, *Siglech*); and (2) non-microglia macrophages (*Lyve1*, *Ly6c1*, *CD163*, *Mrc1*) from each sorting method (AutoMACS, AutoMACS to Tyto, Tyto, and FACSAria) were compared with Cell-Input. ***D***, RNA-Seq gene expression of peripheral macrophage marker genes from (1) infiltrating macrophages (*Emp1*, *Scarf1*, *Emb*, *Clec4e*); and (2) peripheral macrophages/monocytes (*Emilin1*, *F5*, *Fn1*, *Gda*) from each sorting method (AutoMACS, AutoMACS to Tyto, Tyto, and FACSAria) were compared with Cell-Input (one-way ANOVA, Tukey’s *post hoc* test, *p < 0.05). All bars represent the mean normalized gene expression ± SEM. ***E***, GSEA of the 113 microglial marker genes identified in ***A*** comparing all sort positive fractions (AutoMACS, AutoMACS to Tyto, Tyto, and FACSAria) to Cell-Input. ***F***, GSEA of the 174 macrophage marker genes identified in Figure 3*B* comparing all sort positive fractions (AutoMACS, AutoMACS to Tyto, Tyto, and FACSAria) to Cell-Input. ***G***, GSEA enrichment scores compared with Cell-Input for each of the sorting methods for the microglial marker genes identified in ***A*** and macrophage marker genes identified in ***B***.

10.1523/ENEURO.0348-21.2022.f1-3 Extended Data Figure 1-3 Flow cytometry of blood from cre-negative (WT) and Cx3cr1-NuTRAP blood following Tam-mediated cre-recombination. ***A***, ***B***, Gating strategy to assess the level of eGFP-expressing CD11b^+^CD45^+^ singlets (***A***) 3 and (***B***) 60 d following cessation of Tam induction. ***C***, Three days after Tam induction, there is significant expression of the NuTRAP allele in circulating CD11b^+^CD45^+^ cells, which is not evident after 60 d (one-way ANOVA, Tukey’s *post hoc* test, ***p < 0.001). Download Figure 1-3, EPS file.

**Figure 4.**
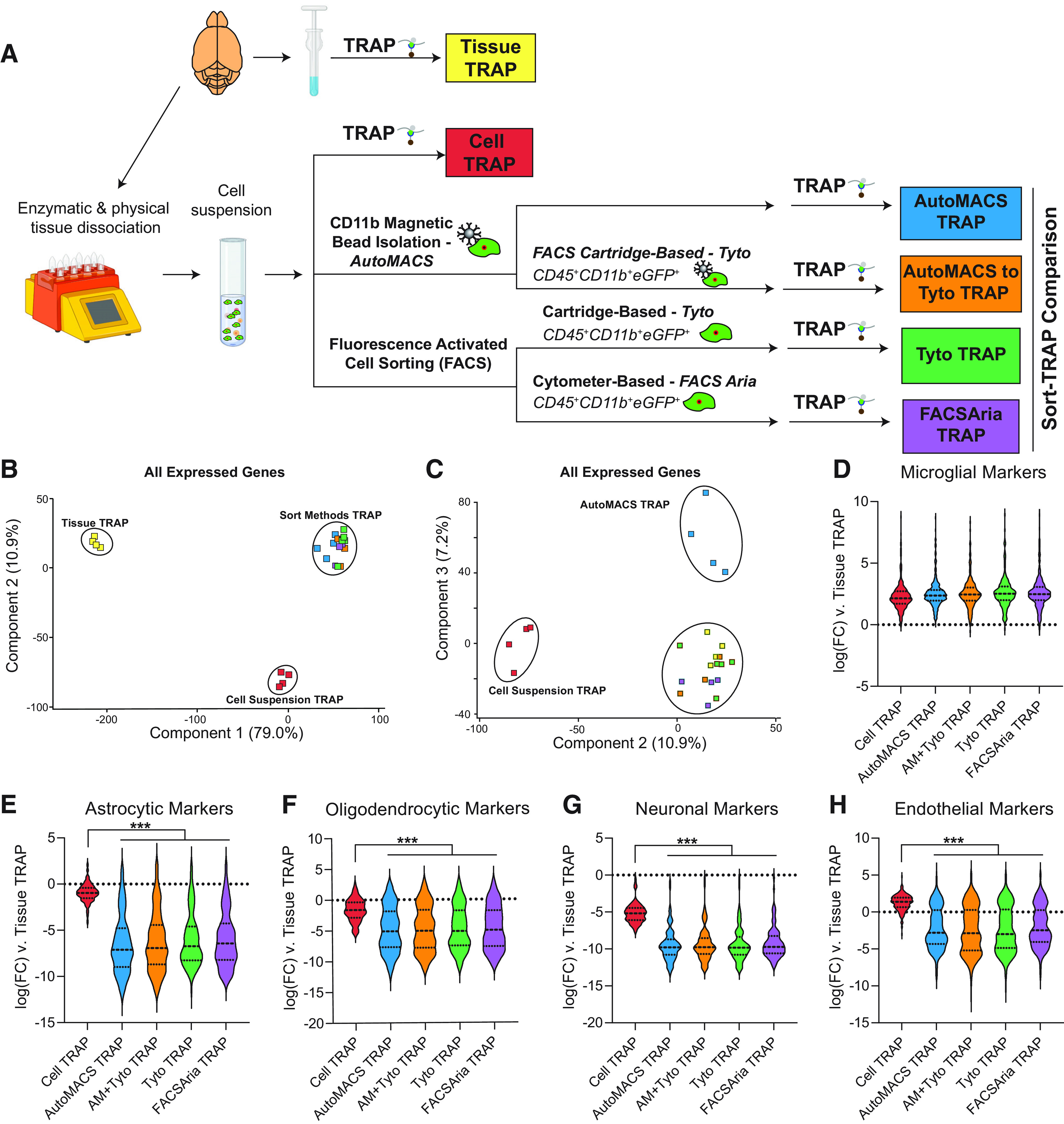
Comparison of TRAP-isolated microglial translatome from tissue homogenate, cell suspension, and various microglial cell sorting methods. ***A***, Schematic of the experimental design. Cx3cr1-NuTRAP brains were hemisected and processed in halves for whole-tissue homogenization or enzymatic and mechanical dissociation to create a single-cell suspension. Single-cell suspensions were then sorted using MACS-based and/or FACS-based isolation of microglia. Tissue homogenate, mixed-cell suspension, and microglia sorted by each of the four depicted methods were subjected to TRAP to isolate microglial-specific ribosomally-bound RNA for creation of RNA-Seq libraries. Gene body coverage plots showed no 3′ bias in any groups (Extended Data Fig. 4-2). ***B***, PCA of all expressed genes (>5 read counts in all samples from at least one group) separates Tissue-TRAP from all other groups in the first component (79% explained variance) and Cell Suspension-TRAP from all other groups in the second component (10.9% explained variance). ***C***, Third component of PCA on all expressed genes separated autoMACS-TRAP from all other groups (7.2% explained variance). Each of the sort strategies had similar levels of (***D***) enrichment of microglial transcripts and depletion of (***E***) astrocytic, (***F***) oligodendrocytic, (***G***) neuronal, and (***H***) endothelial transcripts when compared with Tissue-TRAP. All of the sort methods showed stronger depletion of (***E***) astrocytic, (***F***) oligodendrocytic, (***G***) neuronal, and (***H***) endothelial transcripts when compared with Cell-TRAP (one-way ANOVA, Tukey’s *post hoc* test, ***p < 0.001; Extended Data Fig. 4-1). Created with BioRender.com.

10.1523/ENEURO.0348-21.2022.f1-4 Extended Data Figure 1-4 Flow cytometric analysis of CD45^Mid^ and CD45^High^ cell populations from Cx3cr1-NuTRAP brain Cell-Input and sorted microglia. ***A***, Gating strategy to assess the percent CD45^Mid^ and CD45^High^ of CD11b^+^CD45^+^ singlets. CD45^Mid^ and CD45^High^ populations were then hierarchical gated to assess the percent eGFP^+^ singlets from the CD45^Mid^ and CD45^High^ populations. ***B***, Quantitation of the proportion of CD11b^+^CD45^+^ singlets that were CD45^Mid^ and CD45^High^ from Cell-Input and each of the microglial sorting methods (AM, AM + Tyto, Tyto, FACSAria). ***C***, Quantitation of the proportion of eGFP^+^ singlets within the CD45^Mid^ (left) and CD45^High^ (right) populations (one-way ANOVA, Tukey’s *post hoc* test, ***p < 0.001). Boxplots represent median, Q1, Q3, minimum, and maximum of each dataset. Download Figure 1-4, EPS file.

**Figure 5.**
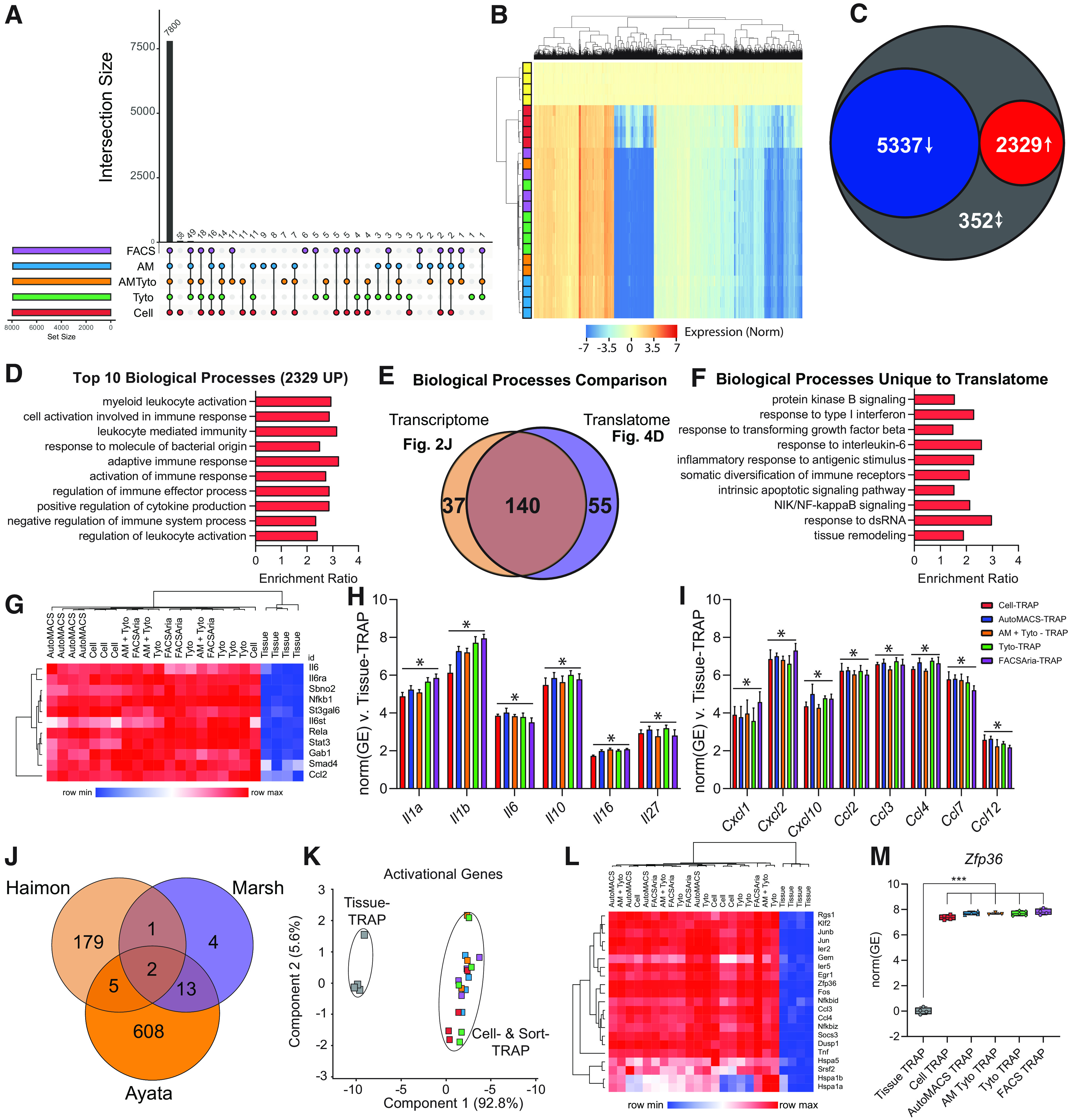
Cell isolation and sorting techniques alter TRAP-isolated microglial translatome compared with whole-tissue TRAP. RNA-Seq libraries were made from each of the groups represented in Figure 3*A* to compare the TRAP-isolated microglial translatomes between whole-tissue-TRAP and each of the cell isolation and sorting methods. All source data for Figure 5 are provided in Extended Data Figure 5-1. ***A***, Upset plot of DEGs for all groups versus Tissue-TRAP (one-way ANOVA, BHMTC, SNK FDR < 0.1, |FC|>2). ***B***, Hierarchical clustering of DEGs shows separation of tissue TRAP from all other groups. Cell-TRAP also clusters separately from all Sort-TRAP groups. ***C***, Comparison of DEGs from each group (Cell- and Sort-TRAP) versus Tissue-TRAP revealed 5337 common DEGs (67%) that were depleted and 2329 common DEGs (29%) that were enriched in all groups (Cell- and Sort-TRAP) compared with Tissue-TRAP. There were only 352 discordant DEGs (4%) between the different sort methods as compared with Cell-Input. ***D***, Top 10 biological processes overrepresented in the 2329 genes upregulated in Cell-TRAP and Sort-TRAP compared with Tissue-TRAP (Gene Ontology ORA, hypergeometric test, BHMTC FDR <0.05). ***E***, Comparison of upregulated transcriptomic pathways (Fig. 2*J*; Extended Data Fig. 2-1) and upregulated translatome pathways (***D***; Extended Data Fig. 5-1) reveal 55 biological processes that are upregulated in the translatome only. ***F***, Selection of 10 biological processes that are uniquely upregulated in the translatome (from the 55 identified in Fig. 4*E*). ***G***, Heatmap of genes involved in “Response to Interleukin-6 (GO:0070741)” biological process. ***H***, Cytokines (*Il1a*, *Il1b*, *Il6*, *Il10*, *Il16*, *Il27*) that are upregulated in the Cell- and Sort-TRAP groups compared with Tissue-TRAP (one-way ANOVA, BHMTC, SNK FDR < 0.1, |FC|>2). ***I***, Chemokines (*Cxcl1*, *Cxcl2*, *Cxcl10*, *Ccl2*, *Ccl3*, *Ccl4*, *Ccl7*, *Ccl12*) that are upregulated in the Cell- and Sort-TRAP groups compared with Tissue-TRAP (one-way ANOVA, BHMTC, SNK FDR < 0.1, |FC|>2). ***J***, Intersection of activational genes identified in three previous studies (Ayata et al., 2018; Haimon et al., 2018; Marsh et al., 2020) identified 21 *ex vivo* activational transcripts represented in at least two of the studies. ***K***, PCA of 21 *ex vivo* activational genes shows clear separation of Tissue-TRAP from all other groups in the first component (92.8% explained variance). ***L***, Heatmap of 21 activational genes shows high levels of *ex vivo* activational transcripts across Cell- and Sort-TRAP methods compared with Tissue-TRAP. ***M***, *Zfp36* is enriched in Cell-TRAP and Sort-TRAP compared with Tissue TRAP (one-way ANOVA, Tukey’s *post hoc* test, ***p < 0.001).

10.1523/ENEURO.0348-21.2022.f1-5 Extended Data Figure 1-5 Cartidge-based FACS gating strategy for microglial sorting on Miltenyi Biotec MACSQuant Tyto. Download Figure 1-5, EPS file.

**Figure 6.**
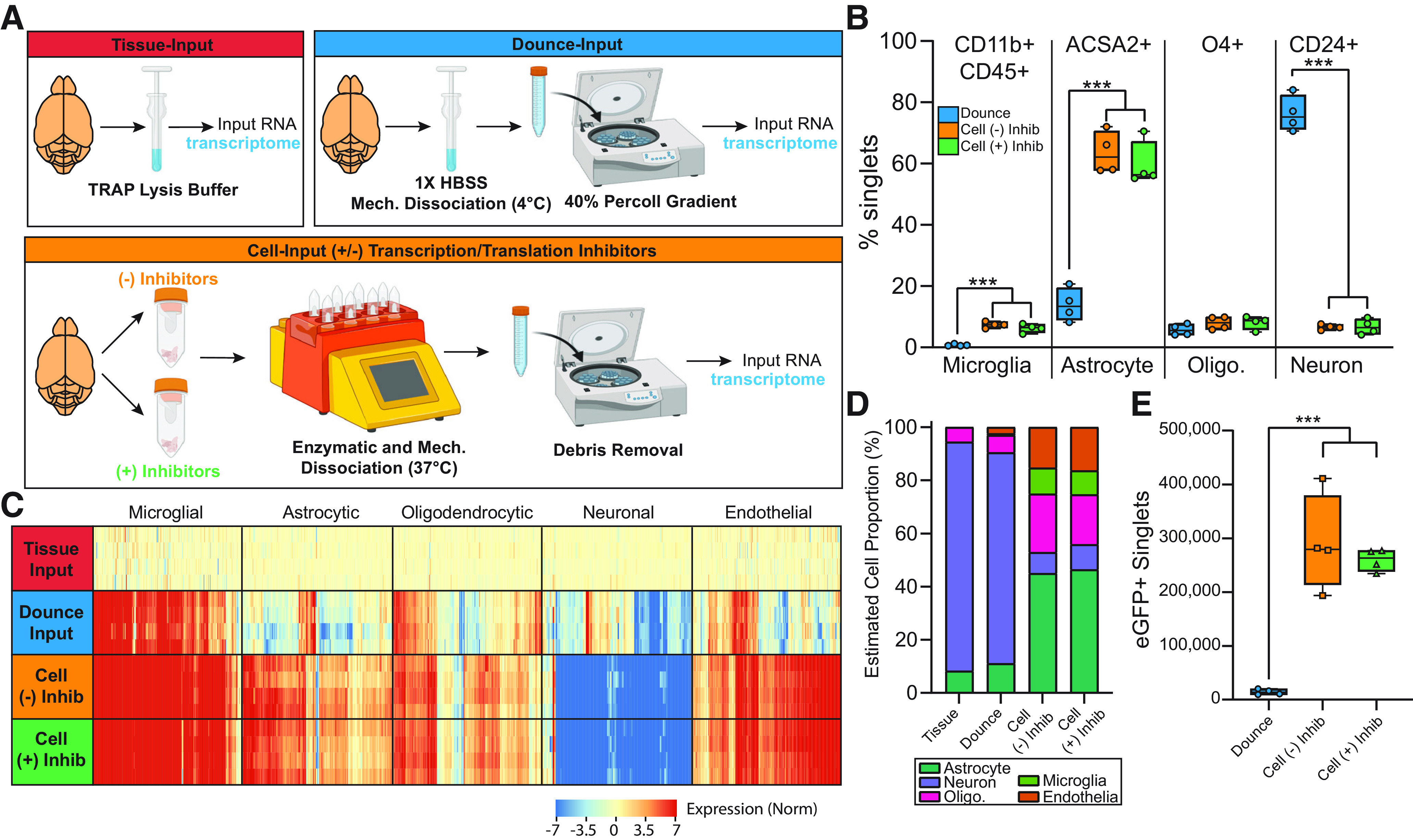
Changes in cellularity based on cell preparation method. Cx3cr1-NuTRAP brains were hemisected and single-cell suspensions were generated by three different methods. Cell preparations were compared via flow cytometry and transcriptomic analyses. The three cell preparation methods were also compared with whole tissue using transcriptomic analysis. Basic sequencing metrics and all source data for Figure 6 are provided in Extended Data Figure 6-1. Gene body coverage plots showed greater 3′ bias in the Tissue- and Dounce-Input groups than the Cell-Input (+/−) Inhibitors groups (Extended Data Fig. 6-2). ***A***, Schematic of experimental design presented in this figure. ***B***, Flow cytometry on cell type-specific markers for microglia (CD11b^+^CD45^+^), astrocytes (ACSA2^+^), oligodendrocytes (O4^+^), and neurons (CD24^+^) for each of the cell preparation methods (one-way ANOVA, Tukey’s *post hoc* test, ***p < 0.001). Representative gating strategies for flow cytometry are provided in Extended Data Figure 6-3. ***C***, Heatmap of cell type-specific markers for microglia, astrocytes, oligodendrocytes, neurons, and endothelial cells. ***D***, CIBERSORTx cellularity estimates based on whole-transcriptome RNA-Seq from whole brain tissue and each of the cell preparation techniques. ***E***, Comparison of the number of eGFP^+^ singlets in each half brain for each of the cell preparation methods (one-way ANOVA, Tukey’s *post hoc* test, ***p < 0.001). There was no overall difference in cell viability between different cell preparation methods. eGFP^+^ cells had slightly higher viability in the Cell-Input (+/−) Inhibitors than the Dounce-Input method (Extended Data Fig. 6-4). Boxplots represent median, Q1, Q3, minimum, and maximum of each dataset. Created with BioRender.com.

10.1523/ENEURO.0348-21.2022.f1-6 Extended Data Figure 1-6 Cytometer-based FACS gating strategy for microglial sorting on FACSAria IIIu. ***A***, Unstained cells from a Cx3cr1-cre/ERT2^Neg^;NuTRAP^+^ (WT) brain were used to establish gating for CD45^+^eGFP^+^CD11b^+^ microglia. ***B***, Gating strategy for Cx3cr1-NuTRAP brain cells stained with CD45-VioBlue and CD11b-APC to sort CD45^+^eGFP^+^CD11b^+^ microglia. Download Figure 1-6, EPS file.

10.1523/ENEURO.0348-21.2022.f1-7 Extended Data Figure 1-7 Gating strategy for assessment of microglial purity by different sort methods. Download Figure 1-7, EPS file.

### Flow cytometric analysis of sort fractions from various microglial sorting techniques

After reserving an aliquot as input, cells were subjected to one of four isolation techniques: (1) CD11b^+^ magnetic-bead based isolation (autoMACS); (2) Cartridge-based FACS on CD11b^+^CD45^+^eGFP^+^ (MACSQuant Tyto); (3) Cytometer-based FACS on CD11b^+^CD45^+^eGFP^+^ (FACSAria); and (4) autoMACS debulking of cells before cartridge-based FACS (autoMACS to MACSQuant Tyto; Fig. 1*A*).

Aliquots of cell input and positive sort fractions from each of the four sort methods were analyzed by flow cytometry. All sort methods showed enriched populations of eGFP^+^ and CD11b^+^CD45^+^ singlets in their positive fractions as compared with Cell-Input (Fig. 1*B*). A leftward shift in CD11-APC was evident in autoMACS samples, likely from competition from the CD11b microbeads used in that isolation. The positive fractions of all sort methods were enriched in eGFP^+^ singlets as compared with the input fraction (Fig. 1C; Extended Data Fig. 1-1; two-way ANOVA, main effect of sort fraction, ***p < 0.001). The autoMACS sort resulted in lower overall percentage of eGFP^+^ singlets compared with all other sort methods and the FACSAria sort resulted in a higher overall percentage of eGFP^+^ singlets, though all approaches demonstrated a high level of enrichment (Fig. 1*C*; Extended Data Fig. 1-1; two-way ANOVA, Tukey’s *post hoc* test, *p < 0.05). The positive fractions of all sort methods were enriched in CD11b^+^CD45^+^ singlets as compared with the input fraction (Fig. 1*D*; Extended Data Fig. 1-1; two-way ANOVA, Main effect of sort fraction, *p* < 0.001). FACSAria sort resulted the highest overall percentage of CD11b^+^CD45^+^ singlets (Fig. 1*D*; Extended Data Fig. 1-1; two-way ANOVA, Tukey’s *post hoc* test, *p < 0.05). Although FACSAria had higher microglial purity than other sort methods, it showed a significantly lower yield than the MACSQuant Tyto sort (Fig. 1*E*; Extended Data Fig. 1-1; one-way ANOVA, Tukey’s *post hoc* test, #*p* < 0.05).

### Comparison of transcriptomic profiles of microglia isolated from various sort methods

Following cell preparation and isolation using methods displayed in Figure 1*A*, RNA was isolated from cells for preparation of stranded RNA-Seq libraries. RNA yield and quality by sort method, in addition to basic sequencing metrics are given in Extended Data Figure 2-1. Gene body coverage plots by experimental group are shown in Extended Data Figure 2-2. All sort methods showed similar coverage across the gene body. We first examined enrichment/depletion of previously published microglial, astrocytic, oligodendrocytic, neuronal, and endothelial markers in the transcriptomic profiles (Chucair-Elliott et al., 2020; McKenzie et al., 2018; Extended Data Fig. 2-1). Each of the four sort methods showed similar levels of enrichment of microglial marker genes (Fig. 2*A*) and depletion of astrocytic, oligodendrocytic, neuronal, and endothelial marker genes (Fig. 2*B–E*) when compared with Cell-Input. In combination with the flow cytometric data presented in Figure 1, this gives confidence that each of the sort methods are effective in isolating highly pure populations of microglia.

10.1523/ENEURO.0348-21.2022.f2-1 Extended Data Figure 2-1 Figure 2 sequencing metrics and source data. Download Figure 2-1, XLS file.

10.1523/ENEURO.0348-21.2022.f2-2 Extended Data Figure 2-2 Gene body coverage plots by sort fraction for sequencing libraries described in Figure 2. Download Figure 2-2, EPS file.

Next, we examined the transcriptomic data in an unbiased manner. PCA of all expressed genes (more than five counts in all samples from at least one group) showed clear separation of Cell-Input from all sort methods in the first component with 81% of the explained variance (Fig. 2*F*). Differentially expressed genes were called by one-way ANOVA with BHMTC followed by Student–Newman–Keuls *post hoc* test (FDR < 0.1, |FC|>2; Extended Data Fig. 2-1). Hierarchical clustering of the 7378 differentially expressed genes (DEGs) shows separation of Cell-Input from all sort methods with similar patterning of enrichment and depletion of DEGs across all sort methods scaled to Cell-Input (Fig. 2*G*). The majority of pairwise DEGs (sort method vs Cell-Input) were in common between all sort methods (7084/7378 = 96%; Fig. 2*H*), suggesting a high degree of similarity between each of the sort methods. In addition, 5322 DEGs (72%) that were down and 1759 DEGs (24%) were up in all sort methods compared with Cell-Input. There were only 297 discordant DEGs (4%) between the different sort methods as compared with Cell-Input (Fig. 2*I*).

ORA of gene ontology (ORA GO) of the 1759 genes that were up across all sort methods identified 177 overrepresented biological processes pathways (BHMTC, FDR < 0.05; Extended Data Fig. 2-1). The “enrichment ratio” is the number of observed genes divided by the number of expected genes from the given gene list. Examination of the top 10 overrepresented biological processes reveals several pathways involved in microglial function, including cytokine-mediated signaling, immune response activation, and adaptive immune response, among others (Fig. 2*J*). Running a similar ORA GO on the 5322 genes down across all sort methods (compared with Cell-Input) revealed 252 overrepresented biological processes (BHMTC, FDR < 0.05; Extended Data Fig. 2-1). The top 10 processes include many neuron-focused pathways, such as neurotransmitter transport, neurotransmitter level regulation, and membrane potential regulation (Fig. 2*K*), indicating depletion of these genes in the sorted cells.

Next, we examined the common 1759 upregulated and 5322 downregulated genes across the four sort methods for overrepresentation of transcription factor targets. Network ORA on the 1759 genes enriched in the positive fraction of all sort methods identified 21 overrepresented transcription factor targets, including the top five hits: ELF1, ETS2, IRF, PU.1, and NFKB (Fig. 2*L*; Extended Data Fig. 2-1). Three of the top five transcription factor targets (ELF1, ETS2, PU.1) are part of the ETS family of transcription factors that assist in regulating immunity (Gallant and Gilkeson, 2006), with PU.1 being a “master regulator” of microglial identity and function (Yeh and Ikezu, 2019). The other two transcription factors (IRF and NFKB) are also critical regulators of inflammation and antiviral response (Iwanaszko and Kimmel, 2015), an important function of microglia. The IRF_Q6 transcription factor motif contains IRF1-regulated genes with 3′ untranslated regions (UTRs) containing the motif BNCRSTTTCANTTYY.

Network ORA of the 5322 genes down in all sort methods compared with input identified 468 overrepresented transcription factor targets, including top five hits: AP-1, TATA, LEF1, PAX4, and AP-4. LEF1 is an endothelium-specific transcription factor (Hupe et al., 2017). AP-4 is an adaptor protein complex that is involved in vesicular trafficking of membrane proteins. Lack of AP-4 has been shown to cause accumulation of axonal autophagosomes containing AMPA receptor components in hippocampal neurons and cerebellar Purkinje cells (Matsuda et al., 2008). Overall, the top transcription factor targets of the genes depleted in the sort fractions (compared with Cell-Input) modulate important non-microglial cellular functions and, as such, are depleted in purified microglia.

In combination, the flow cytometric data, distribution of marker gene enrichments/depletion, and analysis of differentially expressed genes (including pathway and transcription factor analysis) suggest that each of the sort methods are producing highly pure populations of microglia with very similar transcriptomic profiles.

### Flow cytometric analysis of blood from Cx3cr1-NuTRAP mice following Tam-mediated cre recombination

In addition to microglia, the Cx3cr1-cre/ERT2 also targets circulating and peripheral CX3CR1^+^ cells for Tam-mediated cre recombination (Yona et al., 2013) and concomitant expression of the NuTRAP allele. Without perfusion, there is a concern that contamination of circulating CX3CR1^+^ cells may be present in eGFP sorted cells and in TRAP-isolated RNA from Cx3cr1-NuTRAP brain tissue. However, the half-life of circulating CX3CR1^+^ cells in mice is <3 d (Yona et al., 2013) and, accordingly, loss of eGFP-labeling in blood samples from Cx3cr1-NuTRAP mice is evident as CX3CR1^+^ cells are rapidly turned over following Tam induction. To demonstrate the loss of eGFP^+^ in circulating CX3CR1^+^ cells, we collected blood from Cx3cr1-NuTRAP mice 3 and 60 d after cessation of Tam administration and compared the levels of CD11b^+^CD45^+^ cells expressing eGFP to that of cre-negative (WT) controls. There was a marked increase in CD11b^+^CD45^+^eGFP^+^ cells 3 d after Tam induction, which decreased to levels indistinguishable from WT controls after 60 d (Extended Data Fig. 1-3). As such, brains were collected from Cx3cr1-NuTRAP mice at least 60 d following Tam treatment. In studies without transgenic labeling, brains can be perfused with ice-cold physiological saline to eliminate contaminating cells from circulation.

### Assessment of relative levels of non-microglial macrophages within different sort fractions from Cx3cr1-NuTRAP brains

Perivascular, choroid plexus, and meningeal macrophages also express *Cx3cr1* (Faraco et al., 2017), and thus are labeled by the NuTRAP allele (including eGFP-labeled polysomes) on Tam-mediated cre recombination. CD45 expression levels are often used as a FACS sort criteria to separate microglia (CD45^Mid^) from non-microglial macrophages (CD45^High^; Haimon et al., 2018; Marschallinger et al., 2020; Pan and Wan, 2020). The relative levels of non-microglial, macrophage contamination was first assessed by flow cytometry. Cells from each group shown in Figure 1*A* were stained with CD11b and CD45 fluorescent antibodies and analyzed on a MACSQuant Analyzer 10. The percent CD45Mid and CD45High of CD11b^+^CD45^+^ singlets were assessed according to the gating strategy displayed in Extended Data Figure 1-4*A*. The Cell-Input fraction had the highest (16%) CD45^high^ population of CD11b^+^CD45^+^ cells, as compared with the autoMACS (8%), autoMACS to Tyto (5%), Tyto (4%), and FACSAria (6%; Extended Data Fig. 1-4*B*). There was not a significant difference in the proportion of CD45^Mid^ or CD45^High^ (out of CD11b^+^CD45^+^) cells by sort method. The vast majority (>95%) of CD45^Mid^ cell populations co-expressed eGFP regardless of sort method. In contrast, only ∼74% of CD45^High^ cells from the Cell-Input and autoMACS sort fraction co-expressed eGFP. The remaining 26% eGFP^-^CD45^High^ cells are likely contaminating cells from blood circulation. Since the FACS-based methods used eGFP as sorting criteria, the CD45^High^ cells co-expressing eGFP in the cartridge-based Tyto (96%) and cytometer-based FACSAria (99%) were higher than the Cell-Input (Extended Data Fig. 1-4*C*).

Many recent studies have examined differences in the transcriptomic signatures of microglia and non-microglial brain resident macrophages. To further refine our microglial marker list constructed based off a meta-analysis of cell sorting studies (McKenzie et al., 2018), we compared previously published microglial marker lists from cell sorting (Chucair-Elliott et al., 2020), ribosomal tagging (Chucair-Elliott et al., 2020), and a meta-analysis distinguishing microglia from peripheral monocytes and macrophages (Haage et al., 2019). There were 113 genes that were identified as microglial markers in at least two of the three gene lists (highlighted purple) and were used for further analysis (Fig. 3*A*; Extended Data Fig. 3-1). A list of macrophage marker genes was constructed by taking the union of three previously published studies from infiltrating macrophages (DePaula-Silva et al., 2019), peripheral macrophages and monocytes (Haage et al., 2019), and CNS-associated macrophages (CAMs; Faraco et al., 2017) to identify 174 macrophage-specific genes (highlighted green; Fig. 3*B*; Extended Data Fig. 3-1). It is of note that there is no overlap between the 113 microglial-specific genes identified in Figure 3*A* and the 174 macrophage marker genes identified in Figure 3*B*.

10.1523/ENEURO.0348-21.2022.f3-1 Extended Data Figure 3-1 Figure 3 source data. Download Figure 3-1, XLS file.

To increase the specificity of microglial transgenic labeling approaches, recent models such as the Hexb-cre/ERT2 (Masuda et al., 2020a), Tmem119-cre/ERT2 (Kaiser and Feng, 2019), and P2ry12-cre/ERT2 (McKinsey et al., 2020) are more specific to microglia and do not label other CAMs. Siglec-H has also been demonstrated as a discriminating mark on microglia that is absent in CAMs (Konishi et al., 2017). All of these microglial-specific marker genes (*Hexb*, *Tmem119*, *P2ry12*, and *Siglech*) were enriched in the sort fractions when compared with Cell-Input (Fig. 3*C*; Extended Data Fig. 3-1); however, there were no differences in expression between the various sort methods. Next, we examined four marker genes of CAMs (*Lyve1*, *Ly6c1*, *CD163*, and *Mrc1*; Faraco et al., 2017) that are not expressed by microglia. *Lyve1* was enriched in the autoMACS to Tyto sort fraction, *CD163* was enriched in the autoMACS to Tyto and Tyto sort fractions, and *Mrc1* was enriched in all sort fractions as compared with Cell-Input, whereas *Ly6c1* was depleted across all sort methods compared with Cell-Input. Based on this analysis, the sort fractions contain primarily microglia with a small proportion of CAMs present. Separation of microglia and CAMs can be achieved by adding more specific markers to the cell sorting criteria. For the purpose of this study, we will refer to the sort fraction as “microglia.” Overall, markers of infiltrating macrophages (*Emp1*, *Scarf1*, and *Emb*; DePaula-Silva et al., 2019) and peripheral macrophages/monocytes (*Emilin2*, *F5*, *Fn1*, *Gda*) were depleted in the sort fractions when compared with the Cell-Input (Fig. 3*D*; Extended Data Fig. 3-1).

GSEA of the 113 microglial-specific genes identified in Figure 3*A* and the 174 macrophage marker genes identified in Figure 3*B* revealed overall enrichment of microglial marker genes (Fig. 3*E*) and depletion of macrophage marker genes (Fig. 3*F*) when comparing the combined sort fractions from all methods to the Cell-Input. GSEA enrichment scores were similar between the different sort methods (Fig. 3*G*; Extended Data Fig. 3-1). Based on the flow cytometric and transcriptomic data, all sort methods have high levels of microglial purity and low levels of CAM and monocyte contamination.

### Comparison of TRAP-isolated microglial translatome from tissue homogenate, cell suspension, and various microglial sort methods

A schematic of the experimental design is represented in Figure 4*A* Cx3cr1-NuTRAP brains were hemisected and processed in halves for whole-tissue homogenization or enzymatic and mechanical dissociation to create single-cell suspensions. Single-cell suspensions were then sorted using one of four methods: (1) CD11b magnetic-bead based isolation (autoMACS); (2) cartridge-based FACS on CD11b^+^CD45^+^eGFP^+^ (MACSQuant Tyto); (3) cytometer-based FACS on CD11b^+^CD45^+^eGFP^+^ (FACSAria); or (4) autoMACS debulking of cells before cartridge-based FACS (autoMACS to MACSQuant Tyto), as before. Tissue homogenate (Tissue-TRAP), mixed-cell suspension (Cell-TRAP), and sorted microglia (Sort-TRAP) were then subjected to TRAP pull-down of microglial-specific translating RNA for creation of RNA-Seq libraries. RNA yield and quality by sort method, in addition to basic sequencing metrics are given in Extended Data Figure 4-1. Gene body coverage plots by experimental group are shown in Extended Data Figure 4-2. All sort methods showed similar coverage across the gene body.

10.1523/ENEURO.0348-21.2022.f4-1 Extended Data Figure 4-1 Figure 4 sequencing metrics and source data. Download Figure 4-1, XLS file.

10.1523/ENEURO.0348-21.2022.f4-2 Extended Data Figure 4-2 Gene body coverage plots by sort fraction for sequencing libraries described in Figure 4. Download Figure 4-2, EPS file.

PCA of all expressed genes showed separation of Tissue-TRAP from all other groups in the first component with 79% of the explained variance and separation of Cell-TRAP from all other groups with 11% of the explained variance (Fig. 4*B*). The autoMACS-TRAP group separated from the other groups in the third component with 7% of the explained variance (Fig. 4*C*). Based on this unbiased dimensionality reduction, it appears that the MACS-based and FACS-based sorted cells have similar translatome profiles. Each of the four Sort-TRAP methods showed similar levels of enrichment of microglial marker genes (Fig. 4*D*; Extended Data Fig. 4-1). Depletion of astrocytic, oligodendrocytic, neuronal, and endothelial marker genes (Fig. 4*E–H*; Extended Data Fig. 4-1) was greater in Sort-TRAP methods as compared with Cell-TRAP (one-way ANOVA, Tukey’s *post hoc* test, ***p < 0.001). This shows that the extra enrichment step of sorting microglia followed by TRAP-isolation of translating microglial RNA, leads to more pure microglial RNA than Tissue-TRAP or Cell-TRAP alone.

In recent years, several studies have suggested that cell-isolation methods cause *ex vivo* activational effects in microglia (Ayata et al., 2018; Haimon et al., 2018; Marsh et al., 2020). In this section, our goal was to determine whether different sorting techniques result in different levels of *ex vivo* activation. We used Tissue-TRAP as an “unactivated” microglial reference group, since the Tissue-TRAP method does not rely on the creation of a cell suspension or cell sorting techniques. There were 8076 DEGs between the translatomes when Cell- and Sort-TRAP methods were compared with the Tissue-TRAP reference (one-way ANOVA, BHMTC, SNK FDR < 0.1, |FC|>2). Upset plot of the 8076 DEGs shows the majority of the DEGs (7800/8076 = 97%) are in common between all groups (Cell-/Sort-TRAP vs Tissue-TRAP; Fig. 5*A*; Extended Data Fig. 5-1). Hierarchical clustering of all 8076 DEGs shows distinct clustering of Tissue-TRAP from all other groups. Cell-TRAP also clusters separately from the Sort-TRAP groups (Fig. 5*B*; Extended Data Fig. 5-1). Comparison of DEGs from each group (Cell- and Sort-TRAP) versus Tissue-TRAP, revealed 5337 common DEGs (67%) that were depleted and 2329 common DEGs (29%) that were enriched in all groups (Cell- and Sort-TRAP) compared with Tissue-TRAP. There were only 352 discordant DEGs (4%) between the different sort methods as compared with Cell-Input (Fig. 5*C*). These data, together with cell-type enrichments from Figure 4*D–H*, suggests that the act of creating a cell suspension is the largest contributor to differences seen between Tissue-TRAP and Sort-TRAP methods.

10.1523/ENEURO.0348-21.2022.f5-1 Extended Data Figure 5-1 Figure 5 source data. Download Figure 5-1, XLS file.

Next, we identified overrepresented pathways among the upregulated genes compared with Tissue-TRAP. The top 10 biological processes of the 2329 genes upregulated in comparison to Tissue-TRAP were microglial-related pathways, including myeloid leukocyte activation, cell activation involved in immune response, and leukocyte-mediated immunity (Fig. 5*D*; Extended Data Fig. 5-1). Comparing the biological processes identified in the transcriptomic analysis (Fig. 2*J*; Extended Data Fig. 2-1) and the translatomic analysis (Fig. 5*D*; Extended Data Fig. 5-1) revealed 55 biological processes that were only upregulated in the translatome (Fig. 5*E*). Several of the biological processes uniquely upregulated in the translatome comparisons were involved in microglial activation pathways, including response to Type I interferon (IFN), response to transforming growth factor β (TGFB), response to interleukin-6 (IL-6), and NIK/NF-κB signaling (NFKB; Fig. 5*F*). IL-6 is a proinflammatory cytokine extensively studied in brain aging and disease (Ye and Johnson, 1999; Singh-Manoux et al., 2014; Borovcanin et al., 2017). Microglia have higher IL-6 receptor (IL-6R) expression than any other cell type. As such, microglia are highly responsive to IL-6 and transition into a “primed” state when exposed to high levels of IL-6. Hierarchical clustering of the “Response to IL-6” pathway genes showed overall higher levels of expression in Cell- and Sort-TRAP groups (Fig. 5*G*; Extended Data Fig. 5-1). The Cell-TRAP did not cluster separately from the Sort-TRAP groups, providing further evidence that the *ex vivo* activational signature is a function of cell preparation.

Next, we looked at gene expression of common cytokines (Fig. 5*H*; Extended Data Fig. 5-1) and chemokines (Fig. 5*I*; Extended Data Fig. 5-1) across Tissue-, Cell-, and Sort- TRAP groups. We observed higher levels of cytokine and chemokine transcripts across all Cell- and Sort-TRAP groups when compared with Tissue-TRAP (one-way ANOVA, BHMTC, SNK FDR < 0.1, |FC|>2). In an effort to cross-validate our finding with previous studies, we intersected *ex vivo* microglial activational gene lists from three previous studies (Ayata et al., 2018; Haimon et al., 2018; Marsh et al., 2020) and identified 21 *ex vivo* activational transcripts represented in at least two of the studies (Fig. 5*J*; Extended Data Fig. 5-1). PCA of the TRAP data from the present study on the 21 *ex vivo* activational genes shows clear separation of Tissue-TRAP from all other groups in the first component (92.8% explained variance; Fig. 5*K*). Again, suggesting that the *ex vivo* signature is a function of cell preparation. Hierarchical clustering of the 21 activational genes, shows similar patterning as in the “Response to IL-6” pathway with higher levels of expression across Cell- and Sort-TRAP groups compared with Tissue-TRAP (Fig. 5*L*; Extended Data Fig. 5-1). *Zfp36* was one of the *ex vivo* activational genes identified in all three previous studies (Ayata et al., 2018; Haimon et al., 2018; Marsh et al., 2020). Zinc finger protein 36 (*Zfp36*) encodes for the protein tristetraprolin (TTP), which is involved in regulating immune responses through mRNA destabilizing and alternative splicing (Tu et al., 2019). *Zfp36* is enriched in Cell- and Sort-TRAP compared with Tissue TRAP (one-way ANOVA, Tukey’s *post hoc* test, ***p < 0.001; Fig. 5*M*; Extended Data Fig. 5-1).

In summary, these data suggest that *ex vivo* microglial activation is primarily occurring during cell preparation and is sustained through microglial isolation by various sort methods but there were no major differences between the different sort methods.

### Comparison of cellularity and microglial yield between different cell preparation methods

Since *ex vivo* activation appears to be an artifact of cell preparation, three different methods were compared for changes in cellularity within the cell preparation. The experimental design is displayed in Figure 6*A*. Tissue-Input, generated by immediately Dounce homogenizing tissue in TRAP lysis buffer, was used as a comparator to the three methods of cell preparation. The first cell preparation method was a mechanical dissociation conducted entirely at 4°C followed by a 40% Percoll gradient to removed undissociated tissue and myelin debris (Dounce-Input). The second cell preparation method used a papain-based enzyme mixture which requires a brief (30 min) incubation at 37°C followed by myelin debris removal [Cell-Input (−) Inhibitors] which was the method of cell preparation used in Figures 1-5. The third cell preparation method used the same papain-based enzyme mixture, this time supplemented with transcriptional and translation inhibitors (Marsh et al., 2020; Wu et al., 2017) with incubation at 37°C followed by myelin debris removal [Cell-Input (+) Inhibitors]. Cells from each of the three methods were pelleted and lysed with TRAP lysis buffer before RNA extraction and assessment of the transcriptome. RNA yield and quality by sort method, in addition to basic sequencing metrics are given in Extended Data Figure 6-1. The average RNA yield was higher in the Tissue-Input (5.8 μg/half brain) than the Cell-Input (+/−) Inhibitors (360 ng/half brain) and Dounce-Input (35 ng/half brain; one-way ANOVA, Tukey’s *post hoc* test, *p* < 0.05). The average RNA integrity number (RIN) was lowest in the Dounce-Input (3.4) when compared with the Tissue-Input (7.8) and Cell-Input (+/−) Inhibitors (7.3). In addition, use of an entire Cx3cr1-NuTRAP brain was needed for RNA isolation from the Dounce-Input group, whereas half a brain was used for all other methods. Gene body coverage plots by experimental group are shown in Extended Data Figure 6-2. The Tissue-Input and Dounce-Input groups showed more 3′ bias in their gene body coverage than the Cell-Input (+/−) Inhibitor groups.

10.1523/ENEURO.0348-21.2022.f6-1 Extended Data Figure 6-1 Figure 6 sequencing metrics and source data. Download Figure 6-1, XLS file.

10.1523/ENEURO.0348-21.2022.f6-2 Extended Data Figure 6-2 Gene body coverage plots by sort fraction for sequencing libraries described in Figure 6. Download Figure 6-2, EPS file.

10.1523/ENEURO.0348-21.2022.f6-3 Extended Data Figure 6-3 Gating strategy for assessment of cellularity. Download Figure 6-3, EPS file.

To assess the relative cellularity between each of the cell preparation methods, flow cytometry was conducted on each of the cell preparations, using markers for microglia (CD11b^+^CD45^+^ or eGFP^+^), astrocytes (ACSA2^+^), oligodendrocytes (O4^+^), and neurons (CD24^+^). After gating on cells and singlets (as in Extended Data Fig. 6-3) the percent singlets positive for each of the cell markers were compared between methods. There was a higher percentage of neurons and lower percentage of microglia and astrocytes in the Dounce-Input samples as compared with the Cell-Input (+/−) Inhibitors (Fig. 6*B*). There was no overall difference in cell viability between the three cell preparation methods examined. However, there was higher viability among eGFP^+^ singlets in the Cell-Input (+/−) Inhibitors as compared with the Dounce-Input. Inclusion of transcription and translation inhibitors during enzymatic and mechanical dissociation did not alter cell viability (Extended Data Fig. 6-4).

Stranded RNA-Seq libraries were constructed from each of the groups displayed in Figure 6*A*. Heatmaps of microglial, astrocyte, oligodendrocytic, neuronal, and endothelial cell marker lists for each of the cell preparation methods were normalized to Tissue-Input. All three cell preparation methods appear to be enriched for microglial transcripts as compared with Tissue-Input, with the strongest enrichment in the Cell-Input (+/−) Inhibitors. The Cell-Input (+/−) Inhibitors also appear to have greater enrichment of astrocytic and endothelial markers and depletion of neuronal transcripts when compared with Tissue- and Dounce-Input (Fig. 6*C*; Extended Data Fig. 6-1). CIBERSORTx, or digital cytometry, estimates cell-type abundance from bulk transcriptomics. Using publicly available cell-type specific data from mouse brain (Zhang et al., 2014; GSE52564) as a digital cytometry reference matrix, we estimated the cellularity from each of the four groups (Zhang et al., 2014). Based on this analysis, there was no difference in the cellularity between the Tissue-Input and Dounce-Input or between the Cell-Input (−) Inhibitors and the Cell-Input (+) Inhibitors. However, there were more astrocytic, oligodendrocytic, microglial, and endothelial transcripts in the Cell-Input (+/−) Inhibitors than the Tissue- and Dounce-Input. Further, there were less neuronal transcripts in the Cell-Input (+/−) Inhibitors than the Tissue- and Dounce-Input (two-way ANOVA, Tukey’s *post hoc* test; Fig. 6*D*; Extended Data Fig. 6-1). These CIBERSORTx results are consistent with the flow cytometric data displayed in Figure 6*B* and suggest that the enzymatic and mechanical dissociation conducted at partially elevated temperatures depletes neurons from the cell preparation.

Lastly, we compared the cell yield per half brain between the various cell preparation techniques. The average total cell count was slightly higher in the Dounce-Input (∼3.7 × 10^6^ cells/half brain) when compared with Cell-Input (+/−) Inhibitors (∼1.7 × 10^6^ cells/half brain; Extended Data Fig. 6-1). However, because of the increased proportion of microglia in the Cell-Input (+/−) Inhibitors compared with the Dounce-Input, there were ∼20 times more eGFP^+^ microglia in the Cell-Input (+/−) Inhibitors compared with the Dounce-Input (Fig. 6*E*; Extended Data Fig. 6-1). Thus, the enzymatic and mechanical dissociation conducted at partially elevated temperatures will minimize the need for pooling samples when using small tissue sections for microglial sorting applications.

### Effect of different cell preparation techniques on *ex vivo* activational profiles following cell preparation

**Figure 7.**
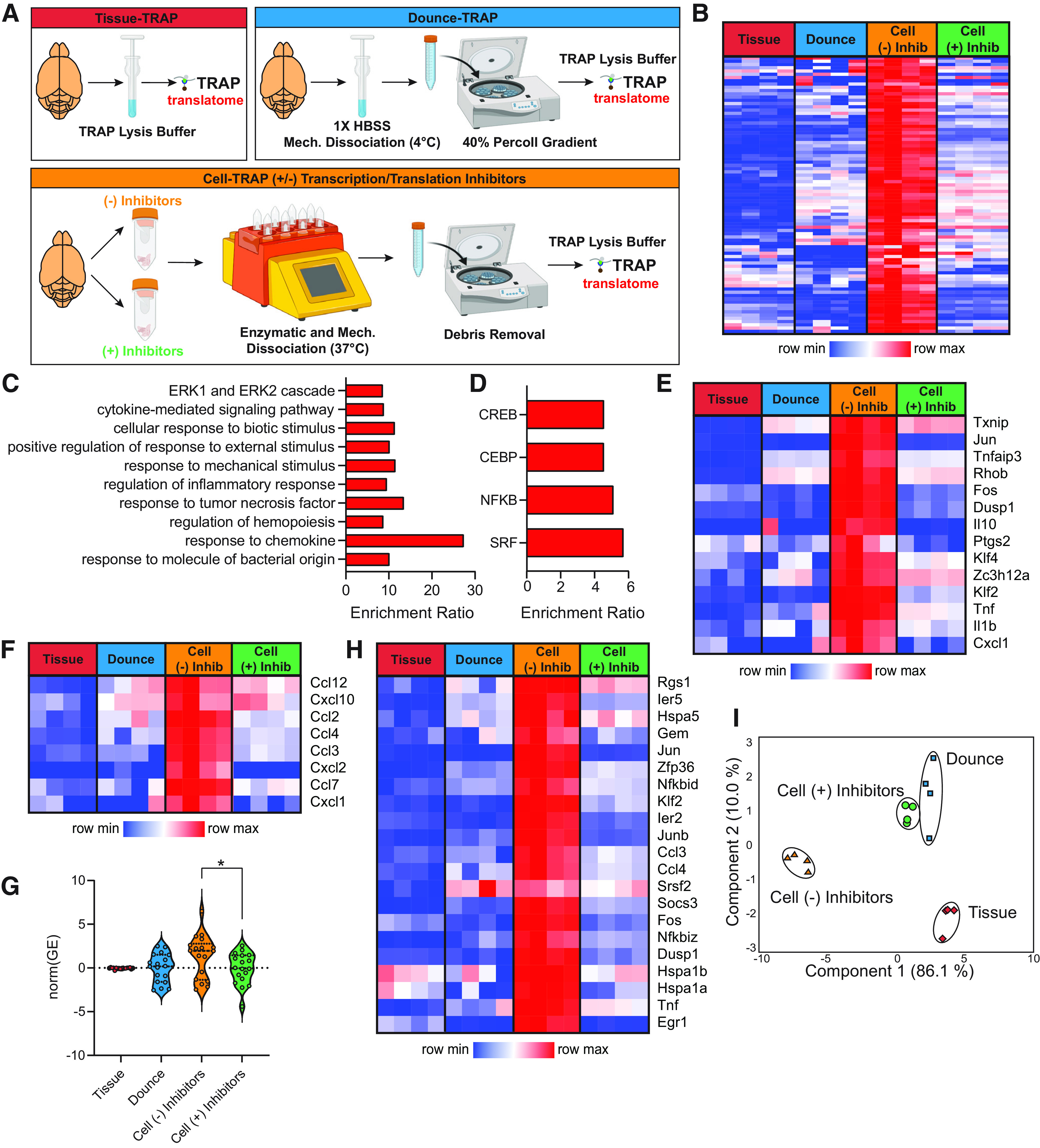
*Ex vivo* activational profiles by cell preparation method. Basic sequencing metrics and all source data for Figure 7 are provided in Extended Data Figure 7-1. Gene body coverage plots showed greater 3′ bias in the Tissue- and Dounce-TRAP groups than the Cell-TRAP (+/−) Inhibitors groups (Extended Data Fig. 7-2). ***A***, Schematic of experimental design presented in this figure. ***B***, Differentially expressed genes were called between Tissue-TRAP, Dounce-TRAP, and Cell-TRAP (+/− Inhibitors) groups (Extended Data Figs. 7-1, 7-3). A subset of genes was activated with cell preparation [Up in Cell-TRAP (−) Inhibitors vs Tissue-TRAP] and were decreased with the addition of inhibitors [Down in Cell-TRAP (+) Inhibitors versus Cell-TRAP (−) Inhibitors; one-way ANOVA, BHMTC, SNK FDR < 0.1, |FC|>2]. These genes were classified as *ex vivo* activational transcripts prevented by the addition of inhibitors and are given in the heatmap. ***C***, Top 10 biological processes overrepresentation in the *ex vivo* activational genes prevented by the addition of inhibitors (hypergeometic test, BHMTC, FDR < 0.05). ***D***, Transcription factors overrepresented in the *ex vivo* activational transcripts prevented by the addition of inhibitors (hypergeometic test, BHMTC, FDR < 0.05). ***E***, Heatmap of select response to oxidative stress pathway genes. ***F***, Heatmap of common chemokines. ***G***, Violin plots of senescent cell marker expression (one-way ANOVA, Tukey’s *post hoc* test, *p < 0.05). ***H***, Heatmap of 21 *ex vivo* activational genes identified in at least two previous studies (Ayata et al., 2018; Haimon et al., 2018; Marsh et al., 2020). ***I***, PCA of 21 *ex vivo* activational genes identified in at least two previous studies (Ayata et al., 2018; Haimon et al., 2018; Marsh et al., 2020).

Recent studies have suggested that inclusion of transcriptional and/or translational inhibitors during cell preparation can prevent *ex vivo* activational confounds in microglia (Marsh et al., 2020) and other neuronal cell types (Wu et al., 2017). Additionally, *ex vivo* artifacts can be prevented by mechanical dissociation conducted entirely at low temperatures (Marsh et al., 2020). To compare the relative levels of *ex vivo* activation between various cell preparation methods, we TRAP-isolated RNA from Tissue, Dounce, and Cell (+/−) Inhibitor cell preparations as shown in Figure 7*A*. Stranded RNA-Seq libraries were constructed from TRAP-isolated translating RNA from each of the four groups. RNA yield and quality by sort method, in addition to basic sequencing metrics, are given in Extended Data Figure 7-1. Gene body coverage plots by experimental group are shown in Extended Data Figure 7-2. The Tissue-TRAP and Dounce-TRAP groups showed more 3′ bias in their gene body coverage than the Cell-TRAP (+/−) Inhibitor groups.

10.1523/ENEURO.0348-21.2022.f7-1 Extended Data Figure 7-1 Figure 7 sequencing metrics and source data. Download Figure 7-1, XLS file.

10.1523/ENEURO.0348-21.2022.f7-2 Extended Data Figure 7-2 Gene body coverage plots by sort fraction for sequencing libraries described in Figure 7. Download Figure 7-2, EPS file.

10.1523/ENEURO.0348-21.2022.f7-3 Extended Data Figure 7-3 Hierarchical clustering of differentially expressed genes between Tissue-TRAP, Dounce-TRAP, Cell-TRAP (−) Inhibitors, and Cell-TRAP (+) Inhibitors. Download Figure 7-3, EPS file.

There were 4475 differentially expressed genes between Tissue-TRAP, Dounce-TRAP, Cell-TRAP (−) Inhibitors, and Cell-TRAP (+) Inhibitors (one-way ANOVA, |FC|>2, BHMTC, FDR < 0.1; Extended Data Figs. 7-1, 7-3). Many of these transcripts differ between all cell preparation methods and the Tissue-TRAP, suggesting the differences may result from slight differences in the purity of TRAP-isolated RNA. There were 85 DEGs that had higher expression in the Cell-TRAP (−) Inhibitors compared with Tissue-TRAP and lower expression in Cell-TRAP (+) Inhibitors compared with Cell-TRAP (−) Inhibitors. These 85 transcripts are *ex vivo* activational transcripts that are prevented by the inclusion of transcriptional and translational inhibitors during enzymatic and mechanical dissociation at partially elevated temperatures. A heatmap of these 85 prevented *ex vivo* activational transcripts showed both the Dounce-TRAP and Cell-TRAP (+) Inhibitors were successful at preventing the induction of *ex vivo* artifacts (Fig. 7*B*; Extended Data Fig. 7-1). The top 10 GO biological processes overrepresented in the 85 prevented *ex vivo* transcripts include ERK1 and ERK2 cascade, cytokine-mediated signaling, cellular response to biotic stimulus, positive regulation of response to external stimulus, response to mechanical stimulus, regulation of inflammatory response, response to tumor necrosis factor, regulation of hemopoiesis, response to chemokine, and response to molecule of bacterial origin (Fig. 7*C*; Extended Data Fig. 7-1). In addition, CREB, CEBP, NFKB, and SRF are overrepresented transcription factors in the 85 prevented *ex vivo* transcripts (Fig. 7*D*; Extended Data Fig. 7-1). Overall, pathway analyses of the 85 prevented *ex vivo* transcripts suggest that mechanical dissociation conducted entirely at chilled temperatures or inclusion of transcription and translation inhibitors during enzymatic and mechanical dissociation at elevated temperatures are successful methods at preventing upregulation of *ex vivo* responsive pathways during cell preparation.

In order to study *in vivo* pathophysiological microglial responses, it is necessary to reduce the *ex vivo* confounds of cell preparation. For example, with aging and disease microglia increase production of inflammatory cytokines, chemokines, and reactive oxygen species (Von Bernhardi et al., 2015). Response to oxidative stress and reactive oxygen species were also identified as pathways significantly overrepresented in the 85 prevented *ex vivo* activational transcripts. Induction of the response to oxidative stress pathway (Fig. 7*E*; Extended Data Fig. 7-1) and generation of common chemokines (Fig. 7*F*; Extended Data Fig. 7-1) were prevented in the Dounce-TRAP and Cell-TRAP (+) Inhibitors as compared with the Cell-TRAP (−) Inhibitors group.

Another active area of research in glial biology is that of cellular senescence, characterized by cell-cycle arrest and secretion of proinflammatory cytokines and chemokines (Hou et al., 2021; Hu et al., 2021). Cell preparation method altered expression of cellular senescence-related genes identified from a previously published study (Aird et al., 2016). Inclusion of transcription and translation inhibitors during cell preparation reduced the induction of these senescence-associated genes (Fig. 7*G*; Extended Data Fig. 7-1).

Heatmap of the 21 *ex vivo* activational transcripts identified in at least two of the three examined previous studies (Ayata et al., 2018; Haimon et al., 2018; Marsh et al., 2020) from Figure 5*J* shows prevention of *ex vivo* activation with the addition of transcriptional and translational inhibitors or mechanical dissociation conducted entirely at low temperatures (Fig. 7*H*; Extended Data Fig. 7-1). PCA of the 21 *ex vivo* activational transcripts identified shows strong separation of Cell-TRAP (−) Inhibitors from all other groups in the first component (86.1% explained variance) and clustering of Dounce-TRAP and Cell-TRAP (+) Inhibitors together (Fig. 7*I*).

In summary, the addition of transcriptional and translational inhibitors during enzymatic and mechanical dissociation of brain tissue or mechanical dissociation conducted entirely at low temperatures can prevent *ex vivo* activational artifacts in microglia.

## Discussion

Microglia have emerged as key players in brain disease, including age-related neuroinflammation and neurodegeneration (Colonna and Butovsky, 2017; Lana et al., 2021). As a minority cell population in the brain, in depth microglial molecular and biochemical analyses benefit from enrichment strategies to provide cell-specific data (Okaty et al., 2011). While *in vitro* cell culture models are useful for mechanistic studies, they fail to recapitulate the complexity of the nervous system milieu. As such, models and methods to “debulk” microglia from brain tissue have become a focus in the field (Chucair-Elliott et al., 2020; McKinsey et al., 2020). The most common methods for isolating microglia include enzymatic and/or mechanical dissociation of brain tissue followed by immunolabeling with magnetic beads for MACS (Bordt et al., 2020) or fluorescent-conjugated antibodies for FACS-based enrichment (Bohlen et al., 2019). As these methods continue to evolve, quantitative comparisons of cell preparation and sort strategies are needed to aid decision-making of what approaches to use in specific studies. As well, there are legitimate concerns that these isolation approaches introduce artifacts, especially in glial cell populations which by nature are sensitive to changes in their microenvironment (Wu et al., 2017). Determining the degree of *ex vivo* activational artifacts, and how they may vary between isolation approaches, has been challenging because the field lacked a resting cell type-specific reference (absent of *ex vivo* activational confounds related to enzymatic/mechanical dissociation) as a comparator.

To address this barrier to progress, we used a ribosomal-tagging model (NuTRAP) in combination with a microglial-specific cre (Cx3cr1-cre/ERT2) to generate a microglial signature without the confounds of cell isolation. We then compared relative microglial enrichment and *ex vivo* activational artifacts between multiple MACS-based and FACS-based cell isolation techniques. All sort methods were successful in isolating highly pure microglia, as evidenced by flow cytometric and transcriptomic analyses. Our MACS-FACS comparison is in line with previous findings on yield and speed (Pan and Wan, 2020). Magnetic bead-based isolation produced nearly as pure of a microglial population as FACS-based approaches. The advantages of the magnetic bead isolation include the rapid isolation (<1 h), multiplexing 6 samples at a time, and least amount of instrumentation. The limitation of magnetic bead-based approaches is the single dimension of labeling as compared with FACS.

Cartridge-based FACS is a new iteration of FACS and produced nearly equivalent cell purities as traditional cytometry-based FACS but with greater cell yield. Given the higher cell yield, cartridge-based FACS may be beneficial for applications requiring large amounts of starting material, including ChIP-seq, HiC-based techniques, proteomics, and primary microglial cultures. Another advantage of this cartridge-based approach is the self-contained nature of the system that does not produce aerosols thus not requiring biosafety containment (Roberts et al., 2021). Cytometry-based FACS produced the purest cell population and has the highest capabilities for dimensions of labeling. However, this approach is also the slowest and had the lowest cellular yield. A strict comparison of FACS methods relies on details of the gating strategies used and these can likely be tuned to emphasize highest purity or cell yield. Cytometer-based FACS easily incorporates various staining controls, which allows for detailed refinement of gating without additional cost. For cartridge-based FACS, separate cartridges are needed for staining controls, increasing the cost. Additionally, the cartridge-FACS does not have traditional forward scatter detection, but instead utilizes back scatter, making it difficult to gate cell populations from debris. Validation of cartridge-FACS sorted cells should be performed on a traditional cytometer as a control.

Combining MACS with cartridge-based FACS, to initially de-bulk microglia and then further purify, did not result in higher purity of microglia and the presence of the magnetic beads shifted signals in the FACS. Taken together, these data demonstrate that all of these approaches are valid for microglial isolation from brain and return highly pure cell populations that are suitable for molecular and biochemical analyses.

An unexpected finding of the analyses was the shift in cellularity caused by the cell suspension preparation. Neuronal cells and transcriptomic signals were depleted during cell preparation with enzymatic methods. While this has the effect of aiding microglial isolation by diminishing the majority neuronal cell population, this has profound effects on neuronal cell isolation studies. Mechanical dissociation at low temperature retained a much higher proportion of neuronal cells. Outside of these studies, others have also observed that alternative cell preparation methods cause less neuronal cell loss (Saxena et al., 2012). We did not test alternate cell preparation approaches such as these for their differential effects on microglial *ex vivo* activation and it remains possible that these different methods, through causing less cell death, could lead to less microglial activation. The large neuronal loss during cell preparation may contribute to microglia activation *ex vivo* given that low temperature mechanical dissociation demonstrated lower activational signal, though this is only an association.

Using TRAP isolation of the microglial translatome as a baseline measure of microglia, induction of *ex vivo* activational pathways occurred during enzymatic and mechanical cell preparation and was sustained during microglial isolation, independent of sort method. Compared with previous studies of these activational artifacts (Ayata et al., 2018; Haimon et al., 2018; Marsh et al., 2020) we found a common set of activational markers centered on immediate early genes. This unified set of markers can be used in future studies as markers of artifactual microglial cell activation. More broadly the use of ribosomal tagging methodologies to provide a comparator for optimizing cell preparation could be applied to any relevant organ.

The use of transcriptional and translational inhibitors (Marsh et al., 2020; Wu et al., 2017) during the cell preparation was investigated in the context of acutely isolating cells for immediate use in downstream molecular and biochemical analyses. Addition of transcriptional and translational inhibitors blocked much of the *ex vivo* activational artifacts without otherwise changing the cell phenotype. Whether this is a valid approach for cells that will subsequently be cultured was not assessed. Nonetheless, as studies also delve into microglial heterogeneity at the single-cell level (Stratoulias et al., 2019; Masuda et al., 2020b) inclusion of inhibitors in the preparation and enrichment of microglia can reduce artifacts in these single-cell studies as well (Marsh et al., 2020). Application of transcriptional and translational inhibitors in the context of specific CNS pathologies should be validated on a case-by-case basis, potentially using the same approaches presented here. As an alternative to enzymatic and mechanical dissociation, mechanical dissociation conducted entirely at 4°C also avoids *ex vivo* activation of microglia. However, the cell/RNA yields and RNA integrity associated with mechanical dissociation was much lower than that obtained with enzymatic and mechanical dissociation.

Collectively, our data demonstrate that a variety of microglial isolation methods can be used with equivalent results and tuned to the needs of the specific study. In addition, activational artifacts occur during cell preparation and can be prevented by inclusion of specific inhibitors early in the cell preparation protocol or by avoiding high temperatures. The different cell sorting methods did not show additional activational effects or differences between methods, indicating that concerns over artifacts should not drive isolation method selection. Addition of transcriptional and translational inhibitors during cell preparation reduces *ex vivo* artifacts and is an easily implementable approach to avoid potential confounds.

## Synthesis

Reviewing Editor: Lindsay De Biase, University of California Los Angeles Decisions are customarily a result of the Reviewing Editor and the peer reviewers coming together and discussing their recommendations until a consensus is reached. When revisions are invited, a fact-based synthesis statement explaining their decision and outlining what is needed to prepare a revision will be listed below. The following reviewer(s) agreed to reveal their identity: NONE.

Summary

Authors seek to compare microglial transcriptome & translatome of ex vivo microglia subjected to various sorting techniques. 1) polysome tagging for isolation of microglial specific mRNAs via Cx3cr1-Cre/ERT2+/WT:NuTRAPflox/WT mice. 2) microglia isolated via cartridge-based FACS. 3), microglia isolated via cytometer-based FACS (CD11b+CD45+eGFP+). 4) Microglia isolated via CD11b magnetic labeling & separation. Flow cytometry analyses show high microglial enrichment for all tested methods versus cell input, with varying cell yields. RNAseq and comparing transcriptomic profiles versus input shows similar patterns of enrichment and depletion of transcripts across sorting methods. TRAP-isolated translatomes also show high similarity to those of microglia sorted by FACS. RNAseq reveals expression of microglial activation genes due to cell preparation in similar manners across preparations. Importantly, adding translation and transcription inhibitors during cell preparation seemingly prevents ex vivo activation of microglia with small reduction to cell yields. Overall, authors show isolation methods are comparable in terms of artifacts, which can be easily prevented with inhibitors.

Reviewers agree that the study appears to be well designed and executed. The results and overall conclusions reported by this work are highly consistent with those of previous, already published studies. While this reduces the novelty of the present work, reviewers agree that overall value of the study remains high; microglia isolation procedure is employed in the majority of studies in neuroimmunology, which is an area of intense research. As such, validation of new cell isolation methodologies and improvement of existing isolation strategies is essential for progress in the field. Moreover, the study pinpoints dissociation methods of the tissue rather than the cell sorting itself as the main cause of gene expression patterns reflective of cell activation.

Major concerns:

1. Is the enzymatic and mechanical dissociation performed with the Miltenyi Adult Brain Dissociation Kit carried out partially at elevated temperature? If so, this needs to be described in the methods. This has already been shown in the literature to result in microglial activation during tissue dissociation (compared with mechanical dissociation carried out entirely at 4 degrees). If the all of the various FACS-based isolation procedures involve tissue dissociation at elevated temperature and this is being compared with NuTrap preparations processed entirely with ice cold buffers, the authors need to revise their claim that tissue preparation and dissociation “per se” results in microglial activation. Tissue preparation and dissociation at elevated temperature certainly can result in microglial activation - but dissociation at 4 degres does not. Ideally the authors would add experiments where they use 4 degree tissue processing followed by microglial isolation via one of their FACS approaches. If this is not possible, the authors need to discuss temperature as a critical factor involved in microglial activation and that tissue processing entirely at 4 degrees can be an alternative to translation / transcription inhibitors for preventing activation. A major drawback of dissociation at 4 degrees is lower cell yields. This can be discussed when comparing the pros and cons of different microglial isolation methods.

2. More detail is needed experimental procedures. Particularly for a study that aims to advance the field by revealing optimal microglial isolation procedures, sufficient detail to allow others to replicate their experiments is essential.

2a. How did the authors proceed with respect to male and female samples? Were they pooled together? If not, were the different groups equally sampled with respect to sex? Also, were any sex differences or biases found? This point should be addressed at the beginning of the results section to set-up the rest of the analyses, and assessment of sex differences can be included as supplementary data.

2b. Did the authors need to pool microglia collected from multiple mice for any of RNA-seq libraries generated? Or does each sample represent microglia isolated from one mouse? Please carefully describe the nature of individual samples and any pooling.

2c. Can the authors provide basic sample metrics regarding RNA yield and quality? Please provide this information in a table and comment on whether the different methods of isolation lead to similar results at the level of RNA yield and quality or not.

2d. When using FACS isolation of microglia, did the authors first gate live cells? If so, this should be described. If not, please describe why not.

2e. What is the “enzyme mixture 1”? (line 175)

2f. What buffer was used to collect microglia, after sorting?

2g. Were libraries amplified with PCR prior sequencing? (e.g. with Nugen Ovation kits or similar)? If yes, provide reagent details, and range of PCR cycles used.

2h. The authors state that library sizing was performed; What fragment range was used?

2i. What sequencing depth was used?

2j. Did all the microglial isolation procedures yield similar library QC results? (e.g., 3’bias, clonality). This could also be reported in a table together with info about RNA yield and quality.

2k. Please provide volume of oil/TAM injected daily. How much time elapsed between the last TAM injection and sacrifice/tissue collection?

2l. In the methodology for the GO term over-representation analyses, can the authors succinctly describe what the Enrichment ratio exactly means? It would seem that if it is a gene ratio, scale values should be in decimals. Given the numbers are integers (1, 2, 3, 4,..) perhaps the ratio refers to another type of measure...

3a. It does not appear that any cardiac perfusion with chilled saline was performed prior to tissue processing. Reviewers agree that this is standard practice in the field and assists critically with removing red blood cells and blood born monocytes and also helps to chill the brain quickly and dampen microglial reactivity. Was any cardiac perfusion performed? If so - please describe. If not - why not? If perfusion was not performed - is contamination by macrophages evident either in the FACS sorting (CD45 high cells) or at the level of gene expression? (The authors can look for published genes that are highly and specifically expressed by macrophages). Even if perfusion was performed, there can be contamination from CNS-associated macrophages such as perivascular macrophages, choroid plexus macrophages and meningeal macrophages. These cells will also be targeted in the Cx3cr1-NuTRAP mice and they will also be labeled by the antibodies used for FACS sorting and CD11b bead enrichment. Again - can the authors detect potential contamination from macrophages and does this degree of contamination vary across isolation methods?

3b. Related to this point - in Table 3-1, many genes are listed that are not exclusively microglia specific. Some genes are indicated as upregulated which are found on CNS-associated macrophages but not on resting microglia such as Mrc1 or Ccr2. A more exact definition of microglial-specific genes is needed and a microglial signature should be clarified and demonstrated in the main figures with separate genes shown.

4. The use of transcription and translation inhibitors is presented as the key to obtaining clean transcriptome signatures and less reactive microglia. As this is a key conclusion of the study, more details are needed regarding the use of the transcription and translation inhibitors such that others in the field could replicate the same workflow. Were the inhibitors included in every buffer used, including brain slicing, single cell suspension, centrifuging, staining, post-staining, and sorting/collection tubes? At what final/working concentration were each of the inhibitors used? Did the authors measure ROS production or expression of ROS-associated gene signatures in the cells upon inhibitor treatment? Will use of the inhibitors interfere with obtaining functional/living cells after sorting which could be used for other studies aside from transcriptomics (for culture of acutely isolated microglia for example)? As mentioned in point #1, ideally the authors would be able to compare microglia isolated using elevated temperature and enzymatic digestion in the presence of translation and transcription inhibitors with the gene expression patterns of microglia that were dissociated and isolated entirely at 4 degrees.

5. Reviewers raise several questions with respect to the FACS gating strategies.

5a. Based on Figure 1-2, it would seem that the Cartridge-based method would make it difficult to clearly define the microglial population from debris. Have the authors cross-validated the samples collected from Cartridge-based method with standard flow cytometer analyses to ensure purity? Please provide these data if available. Otherwise, please comment on the advantages and disadvantages of this methodology; it would seem the method may not fare well in pathological settings...

5b. Related to the point above, reviewers suggest the results obtained with the Tyto sorter may represent a game-changer; 300,000 microglia/half adult brain is a high yeild and may facilitate application of other analyses that require relatively large quantity of starting biological material, including but not limited to ChIP-seq, HiC-based techniques, proteomics, and primary microglial cultures. The authors may want to highlight this point in their discussion. Note that this would also potentially benefit many groups that study microglia isolated from the human brain. Given this, comments on this sorting strategy pros and cons (and how accurate or not it is) are even more warranted.

5c. Why did the authors gate only half of the microglial population with the FACSAria (Figure 1-3)? The microglial “cloud” population is clearly definable, yet the EGFP+ gate displayed cuts across it in the middle. Why? This necessarily lowers yield, and may bias what portion of the microglial cell population that this method samples.

6. Ideally the authors would include an inflammatory challenge (e.g. LPS injection, Chucair-Elliott et al., 2020) or isolation of microglia from a disease model to demonstrate that performance of these various isolation approaches is not radically different in the context of CNS pathology. This is relevant because a significant proportion of studies of microglial cell function are investigating these cells in the context of injury or disease. Reviewers agree that addition of this type of data would greatly strengthen the manuscript. The authors would not need to repeat all analyses, but perhaps examine two of the most promising cell isolation methods. Reviewers also agree that including these data may be beyond the scope of the current manuscript but this needs to at least be discussed as a critical future direction.

Minor comments:

1. The Venn diagram for figure 6B is very difficult to interpret. There is a wealth of information displayed in that insert, and a Venn diagram may not be the optimal method to convey a clear message. Please reconsider the plotting options to enhance clarity of the point made. Perhaps a table could also be considered.

2. Please provide numerical values to accompany the colored scale legends of the different heatmaps included in each figure.

3. In lines 363-371, the authors make a rather strong link between AP-1 and neuronal activity. While this is indeed supported by the literature, the wording used make this association appears quite exclusive. However, AP-1 motifs have been shown to be enriched in microglia at their enhancer genomic regulatory elements (Gosselin et al, Science, 2017 PMID 28546318). Furthermore, AP-1 are also important to the regulation of macrophage immune responses (Fonseca et al. Nat Comm., 2020 PMID 30679424). Together, this suggests that there is no privileged relationship between AP-1 and neurons. Please acknowledge this and/or modify the AP-1-neuronal link passage accordingly in the results section.

4. The authors don’t mention that eGFP is a a component of the NuTRAP construct until lines 223-224, yet eGFP is mentioned prior to this. Please briefly describing the NuTRAP construct in the results right away to reduce confusion.

5. Inconsistencies in nomenclature need to be corrected (e.g., Cx3cr1-Cre/ERT2 in line 88 and Cx3cr1-cre/ERT2 in line 90). Cluster of differentiation should be CD not Cd.

6. Please write gene names in italic where possible.

7. Which transcription factor of the Irf family is meant with Irf; there are many which are differentially regulated in microglia?

8. Figure 6h is missing a legend

## Author Response

Dear Dr. Baise:

We are excited to receive acceptance for publication in eNeuro! We appreciate the reviewer critiques of our

manuscript, “Minimizing the ex vivo confounds of cell-isolation techniques on transcriptomic profiles of

purified microglia.” We have updated the text to address the final comments. Changes to the text are indicated

in red. Detailed below are the point-by-point description of the changes.

Synthesis Statement for Author (Required):

The authors have added a number of new experiments and analyses in this revision. They have also made key

changes to the text and figures. Collectively, the reviewers agree that these modifications have greatly

strengthened the manuscript and almost completely addressed reviewer concerns.

Reviewers request 3 small changes to text if possible, before final publication.

1) Regarding the absence of live/dead staining used during the FACS sort. Dead or dying cells from

enzymatic digestion might change the transcriptomic profiling. Furthermore, the used inhibitors

might affect this as well. If possible, can the authors state in the text if the percentage of dead or

damaged cells is changed between different protocols?

We added supplemental Figure 6-3 to compare cell viability between different cell preparation methods. We

found that the overall cell viability was not affected by the cell preparation method. When looking at eGFP+

cells, the viability was significantly higher when using the enzymatic and mechanical dissociation (as

compared to purely mechanical dissociation at 4{degree sign}C). There was no difference in the cell viability with the

addition of transcription and translation inhibitors during enzymatic and mechanical dissociation. We have

updated the methods and results sections to include these data.

2) Please avoid calling CD45+ CD11b+ cells in the blood “monocytes”. With this basic gating strategy

these cells can include a lot of different cell types in the blood, even beyond distinct monocyte

subsets.

We updated the text to avoid calling CD11b+CD45+ cells “monocytes", where appropriate.

3) Regarding text descriptions of Figure 3. There is still potential evidence that there is a small degree

of contamination with CAMs, as seen by the expression of their signature genes. Text could be

adjusted to say that the Cx3cr1-CreERT2 mouse model allows purification of CNS-resident

macrophages (also nicely shown by the authors here how the monocyte and macrophage markers

are depleted) and that the sort mostly includes microglia with a minor contamination of CNS-resident

macrophages. The authors could also mention follow up studies using additional markers in the

FACS approaches to identify microglia, CAMs or monocytes.

We updated the text for Figure 3 to acknowledge potential contamination of CAMs in the sort fractions and

suggest addition of more specific markers to aid in differentiating microglia and CAMs.

## References

[B1] Aird KM, Iwasaki O, Kossenkov AV, Tanizawa H, Fatkhutdinov N, Bitler BG, Le L, Alicea G, Yang TL, Johnson FB, Noma KI, Zhang R (2016) HMGB2 orchestrates the chromatin landscape of senescence-associated secretory phenotype gene loci. J Cell Biol 215:325–334. 10.1083/jcb.201608026 27799366 PMC5100296

[B2] Avignone E, Lepleux M, Angibaud J, Nägerl UV (2015) Altered morphological dynamics of activated microglia after induction of status epilepticus. J Neuroinflammation 12:202. 10.1186/s12974-015-0421-6 26538404 PMC4634193

[B3] Ayata P, Badimon A, Strasburger HJ, Duff MK, Montgomery SE, Loh YHE, Ebert A, Pimenova AA, Ramirez BR, Chan AT, Sullivan JM, Purushothaman I, Scarpa JR, Goate AM, Busslinger M, Shen L, Losic B, Schaefer A (2018) Epigenetic regulation of brain region-specific microglia clearance activity. Nat Neurosci 21:1049–1060. 10.1038/s41593-018-0192-3 30038282 PMC6090564

[B4] Bohlen CJ, Bennett FC, Bennett ML (2019) Isolation and culture of microglia. Curr Protoc Immunol 125:e70. 10.1002/cpim.70 30414379 PMC6510657

[B5] Bordt EA, Block CL, Petrozziello T, Sadri-Vakili G, Smith CJ, Edlow AG, Bilbo SD (2020) Isolation of microglia from mouse or human tissue. STAR Protoc 1:100035. 32783030 10.1016/j.xpro.2020.100035 PMC7416840

[B6] Borovcanin MM, Jovanovic I, Radosavljevic G, Pantic J, Minic Janicijevic S, Arsenijevic N, Lukic ML (2017) Interleukin-6 in schizophrenia—is there a therapeutic relevance? Front Psychiatry 8:221. 10.3389/fpsyt.2017.00221 29163240 PMC5681495

[B7] Butovsky O, Weiner HL (2018) Microglial signatures and their role in health and disease. Nat Rev Neurosci 19:622–635. 10.1038/s41583-018-0057-5 30206328 PMC7255106

[B8] Cahoy JD, Emery B, Kaushal A, Foo LC, Zamanian JL, Christopherson KS, Xing Y, Lubischer JL, Krieg PA, Krupenko SA, Thompson WJ, Barres BA (2008) A transcriptome database for astrocytes, neurons, and oligodendrocytes: a new resource for understanding brain development and function. J Neurosci 28:264–278. 10.1523/JNEUROSCI.4178-07.2008 18171944 PMC6671143

[B60] Chucair-Elliott AJ, Ocanas SR, Stanford DR, Hadad N, Wronowski W, Otalora L, Stout MB, Freeman WM (2019) Tamoxifen induction of Cre recombinase does not cause long-lasting or sexually divergent responses in the CNS epigenome or transcriptome: implications for the design of aging studies. Geroscience 41:691–708. 31493147 10.1007/s11357-019-00090-2 PMC6885072

[B9] Chucair-Elliott AJ, Ocañas SR, Stanford DR, Ansere VA, Buettner KB, Porter H, Eliason NL, Reid JJ, Sharpe AL, Stout MB, Beckstead MJ, Miller BF, Richardson A, Freeman WM (2020) Inducible cell-specific mouse models for paired epigenetic and transcriptomic studies of microglia and astroglia. Commun Biol 3:693. 10.1038/s42003-020-01418-x 33214681 PMC7678837

[B10] Colonna M, Butovsky O (2017) Microglia function in the central nervous system during health and neurodegeneration. Annu Rev Immunol 35:441–468. 10.1146/annurev-immunol-051116-052358 28226226 PMC8167938

[B11] Cuadros MA, Navascués J (1998) The origin and differentiation of microglial cells during development. Prog Neurobiol 56:173–189. 10.1016/s0301-0082(98)00035-5 9760700

[B12] DePaula-Silva AB, Gorbea C, Doty DJ, Libbey JE, Sanchez JMS, Hanak TJ, Cazalla D, Fujinami RS (2019) Differential transcriptional profiles identify microglial- and macrophage-specific gene markers expressed during virus-induced neuroinflammation. J Neuroinflammation 16:152. 10.1186/s12974-019-1545-x 31325960 PMC6642742

[B13] Dubbelaar ML, Kracht L, Eggen BJL, Boddeke EWGM (2018) The kaleidoscope of microglial phenotypes. Front Immunol 9:1753–1753. 10.3389/fimmu.2018.01753 30108586 PMC6079257

[B14] Faraco G, Park L, Anrather J, Iadecola C (2017) Brain perivascular macrophages: characterization and functional roles in health and disease. J Mol Med (Berl) 95:1143–1152. 10.1007/s00109-017-1573-x 28782084 PMC5812456

[B15] Gallant S, Gilkeson G (2006) ETS transcription factors and regulation of immunity. Arch Immunol Ther Exp (Warsz) 54:149–163. 10.1007/s00005-006-0017-z 16652219

[B16] Haage V, Semtner M, Vidal RO, Hernandez DP, Pong WW, Chen Z, Hambardzumyan D, Magrini V, Ly A, Walker J, Mardis E, Mertins P, Sauer S, Kettenmann H, Gutmann DH (2019) Comprehensive gene expression meta-analysis identifies signature genes that distinguish microglia from peripheral monocytes/macrophages in health and glioma. Acta Neuropathol Commun 7:20. 10.1186/s40478-019-0665-y 30764877 PMC6376799

[B17] Haimon Z, Volaski A, Orthgiess J, Boura-Halfon S, Varol D, Shemer A, Yona S, Zuckerman B, David E, Chappell-Maor L, Bechmann I, Gericke M, Ulitsky I, Jung S (2018) Re-evaluating microglia expression profiles using RiboTag and cell isolation strategies. Nat Immunol 19:636–644. 10.1038/s41590-018-0110-6 29777220 PMC5986066

[B18] Hammond TR, Dufort C, Dissing-Olesen L, Giera S, Young A, Wysoker A, Walker AJ, Gergits F, Segel M, Nemesh J, Marsh SE, Saunders A, Macosko E, Ginhoux F, Chen J, Franklin RJM, Piao X, McCarroll SA, Stevens B (2019) Single-cell RNA sequencing of microglia throughout the mouse lifespan and in the injured brain reveals complex cell-state changes. Immunity 50:253–271.e6. 10.1016/j.immuni.2018.11.004 30471926 PMC6655561

[B19] Han J, Fan Y, Zhou K, Blomgren K, Harris RA (2021) Uncovering sex differences of rodent microglia. J Neuroinflammation 18:74. 10.1186/s12974-021-02124-z 33731174 PMC7972194

[B20] Hickman SE, Kingery ND, Ohsumi TK, Borowsky ML, Wang LC, Means TK, El Khoury J (2013) The microglial sensome revealed by direct RNA sequencing. Nat Neurosci 16:1896–1905. 10.1038/nn.3554 24162652 PMC3840123

[B21] Holt LM, Olsen ML (2016) Novel applications of magnetic cell sorting to analyze cell-type specific gene and protein expression in the central nervous system. PLoS One 11:e0150290. 10.1371/journal.pone.0150290 26919701 PMC4769085

[B22] Hou Y, Wei Y, Lautrup S, Yang B, Wang Y, Cordonnier S, Mattson MP, Croteau DL, Bohr VA (2021) NAD^+^ supplementation reduces neuroinflammation and cell senescence in a transgenic mouse model of Alzheimer’s disease via cGAS–STING. Proc Natl Acad Sci USA 118:e2011226118. 10.1073/pnas.2011226118 34497121 PMC8449423

[B23] Hu Y, Fryatt GL, Ghorbani M, Obst J, Menassa DA, Martin-Estebane M, Muntslag TAO, Olmos-Alonso A, Guerrero-Carrasco M, Thomas D, Cragg MS, Gomez-Nicola D (2021) Replicative senescence dictates the emergence of disease-associated microglia and contributes to Aβ pathology. Cell Rep 35:109228. 10.1016/j.celrep.2021.109228 34107254 PMC8206957

[B24] Hupe M, Li MX, Kneitz S, Davydova D, Yokota C, Kele J, Hot B, Stenman JM, Gessler M (2017) Gene expression profiles of brain endothelial cells during embryonic development at bulk and single-cell levels. Sci Signal 10:eaag2476. 10.1126/scisignal.aag2476 28698213

[B25] Iwanaszko M, Kimmel M (2015) NF-κB and IRF pathways: cross-regulation on target genes promoter level. BMC genomics 16:307–307. 10.1186/s12864-015-1511-7 25888367 PMC4430024

[B26] Kaiser T, Feng G (2019) Tmem119-EGFP and Tmem119-CreERT2 transgenic mice for labeling and manipulating microglia. eNeuro 6:ENEURO.0448-18.2019. 10.1523/ENEURO.0448-18.2019 PMC6712208 31371457

[B27] Konishi H, Kobayashi M, Kunisawa T, Imai K, Sayo A, Malissen B, Crocker PR, Sato K, Kiyama H (2017) Siglec-H is a microglia-specific marker that discriminates microglia from CNS-associated macrophages and CNS-infiltrating monocytes. Glia 65:1927–1943. 10.1002/glia.23204 28836308

[B28] Lana D, Ugolini F, Nosi D, Wenk GL, Giovannini MG (2021) The emerging role of the interplay among astrocytes, microglia, and neurons in the hippocampus in health and disease. Front Aging Neurosci 13:651973. 10.3389/fnagi.2021.651973 33889084 PMC8055856

[B29] Liao Y, Wang J, Jaehnig EJ, Shi Z, Zhang B (2019) WebGestalt 2019: gene set analysis toolkit with revamped UIs and APIs. Nucleic Acids Res 47:W199–W205. 10.1093/nar/gkz401 31114916 PMC6602449

[B30] Marsh SE, Kamath T, Walker AJ, Dissing-Olesen L, Hammond TR, Young AMH, Abdulraouf A, Nadaf N, Dufort C, Murphy S, Kozareva V, Vanderburg C, Hong S, Bulstrode H, Hutchinson PJ, Gaffney DJ, Franklin RJM, Macosko EZ, Stevens B (2020) Single cell sequencing reveals glial specific responses to tissue processing & enzymatic dissociation in mice and humans. bioRxiv. doi: 10.1101/2020.12.03.408542.

[B31] Marschallinger J, Iram T, Zardeneta M, Lee SE, Lehallier B, Haney MS, Pluvinage JV, Mathur V, Hahn O, Morgens DW, Kim J, Tevini J, Felder TK, Wolinski H, Bertozzi CR, Bassik MC, Aigner L, Wyss-Coray T (2020) Lipid-droplet-accumulating microglia represent a dysfunctional and proinflammatory state in the aging brain. Nat Neurosci 23:194–208. 10.1038/s41593-019-0566-1 31959936 PMC7595134

[B32] Masuda T, Amann L, Sankowski R, Staszewski O, Lenz M, D Errico P, Snaidero N, Costa Jordão MJ, Böttcher C, Kierdorf K, Jung S, Priller J, Misgeld T, Vlachos A, Meyer-Luehmann M, Knobeloch KP, Prinz M (2020a) Novel Hexb-based tools for studying microglia in the CNS. Nat Immunol 21:802–815. 10.1038/s41590-020-0707-4 32541832

[B33] Masuda T, Sankowski R, Staszewski O, Prinz M (2020b) Microglia heterogeneity in the single-cell era. Cell Rep 30:1271–1281. 10.1016/j.celrep.2020.01.010 32023447

[B34] Matsuda S, Miura E, Matsuda K, Kakegawa W, Kohda K, Watanabe M, Yuzaki M (2008) Accumulation of AMPA receptors in autophagosomes in neuronal axons lacking adaptor protein AP-4. Neuron 57:730–745. 10.1016/j.neuron.2008.02.012 18341993

[B35] McKenzie AT, Wang M, Hauberg ME, Fullard JF, Kozlenkov A, Keenan A, Hurd YL, Dracheva S, Casaccia P, Roussos P, Zhang B (2018) Brain cell type specific gene expression and co-expression network architectures. Sci Rep 8:8868. 10.1038/s41598-018-27293-5 29892006 PMC5995803

[B36] McKinsey GL, Lizama CO, Keown-Lang AE, Niu A, Santander N, Larpthaveesarp A, Chee E, Gonzalez FF, Arnold TD (2020) A new genetic strategy for targeting microglia in development and disease. Elife 9:e54590. 10.7554/eLife.54590 32573436 PMC7375817

[B37] Mootha VK, et al. (2003) PGC-1alpha-responsive genes involved in oxidative phosphorylation are coordinately downregulated in human diabetes. Nat Genet 34:267–273. 10.1038/ng1180 12808457

[B38] Nikodemova M, Watters JJ (2012) Efficient isolation of live microglia with preserved phenotypes from adult mouse brain. J Neuroinflammation 9:147. 10.1186/1742-2094-9-147 22742584 PMC3418565

[B39] Okaty BW, Sugino K, Nelson SB (2011) Cell type-specific transcriptomics in the brain. J Neurosci 31:6939–6943. 10.1523/JNEUROSCI.0626-11.2011 21562254 PMC3142746

[B40] Pan J, Wan J (2020) Methodological comparison of FACS and MACS isolation of enriched microglia and astrocytes from mouse brain. J Immunol Methods 486:112834. 10.1016/j.jim.2020.112834 32810482

[B41] Prinz M, Jung S, Priller J (2019) Microglia biology: one century of evolving concepts. Cell 179:292–311. 10.1016/j.cell.2019.08.053 31585077

[B42] Provenzano F, Pérez MJ, Deleidi M (2021) Redefining microglial identity in health and disease at single-cell resolution. Trends Mol Med 27:47–59. 10.1016/j.molmed.2020.09.001 33008729

[B43] Reichard A, Asosingh K (2019) Best practices for preparing a single cell suspension from solid tissues for flow cytometry. Cytometry A 95:219–226. 10.1002/cyto.a.23690 30523671 PMC6375754

[B44] Roberts MR, Anderson R, Carmody W, Bosio CM (2021) Validation and application of a benchtop cell sorter in a biosafety level 3 containment setting. Appl Biosaf 26:205–209. 10.1089/apb.20.0065 PMC9134337 36034097

[B45] Rock RB, Gekker G, Hu S, Sheng WS, Cheeran M, Lokensgard JR, Peterson PK (2004) Role of microglia in central nervous system infections. Clin Microbiol Rev 17:942–964. 10.1128/CMR.17.4.942-964.2004 15489356 PMC523558

[B46] Roh HC, Tsai LT, Lyubetskaya A, Tenen D, Kumari M, Rosen ED (2017) Simultaneous transcriptional and epigenomic profiling from specific cell types within heterogeneous tissues in vivo. Cell Rep 18:1048–1061. 10.1016/j.celrep.2016.12.087 28122230 PMC5291126

[B47] Salter MW, Stevens B (2017) Microglia emerge as central players in brain disease. Nat Med 23:1018–1027. 10.1038/nm.4397 28886007

[B48] Saxena A, Wagatsuma A, Noro Y, Kuji T, Asaka-Oba A, Watahiki A, Gurnot C, Fagiolini M, Hensch TK, Carninci P (2012) Trehalose-enhanced isolation of neuronal sub-types from adult mouse brain. Biotechniques 52:381–385. 10.2144/0000113878 22668417 PMC3696583

[B49] Sierra A, Abiega O, Shahraz A, Neumann H (2013) Janus-faced microglia: beneficial and detrimental consequences of microglial phagocytosis. Front Cell Neurosci 7:6. 10.3389/fncel.2013.00006 23386811 PMC3558702

[B50] Singh-Manoux A, Dugravot A, Brunner E, Kumari M, Shipley M, Elbaz A, Kivimaki M (2014) Interleukin-6 and C-reactive protein as predictors of cognitive decline in late midlife. Neurology 83:486–493. 10.1212/WNL.0000000000000665 24991031 PMC4141998

[B51] Stratoulias V, Venero JL, Tremblay ME, Joseph B (2019) Microglial subtypes: diversity within the microglial community. EMBO J 38:e101997. 10.15252/embj.2019101997 31373067 PMC6717890

[B52] Subramanian A, Tamayo P, Mootha VK, Mukherjee S, Ebert BL, Gillette MA, Paulovich A, Pomeroy SL, Golub TR, Lander ES, Mesirov JP (2005) Gene set enrichment analysis: a knowledge-based approach for interpreting genome-wide expression profiles. Proc Natl Acad Sci U S A 102:15545–15550. 10.1073/pnas.0506580102 16199517 PMC1239896

[B53] Tu Y, Wu X, Yu F, Dang J, Wang J, Wei Y, Cai Z, Zhou Z, Liao W, Li L, Zhang Y (2019) Tristetraprolin specifically regulates the expression and alternative splicing of immune response genes in HeLa cells. BMC Immunol 20:13. 10.1186/s12865-019-0292-1 31046669 PMC6498542

[B54] Von Bernhardi R, Eugenín-von Bernhardi L, Eugenín J (2015) Microglial cell dysregulation in brain aging and neurodegeneration. Front Aging Neurosci 7:124. 10.3389/fnagi.2015.00124 26257642 PMC4507468

[B55] Wu YE, Pan L, Zuo Y, Li X, Hong W (2017) Detecting activated cell populations using single-cell RNA-seq. Neuron 96:313–329.e6. 10.1016/j.neuron.2017.09.026 29024657

[B56] Ye SM, Johnson RW (1999) Increased interleukin-6 expression by microglia from brain of aged mice. J Neuroimmunol 93:139–148. 10.1016/s0165-5728(98)00217-3 10378877

[B57] Yeh H, Ikezu T (2019) Transcriptional and epigenetic regulation of microglia in health and disease. Trends Mol Med 25:96–111. 10.1016/j.molmed.2018.11.004 30578089 PMC6377292

[B58] Yona S, Kim KW, Wolf Y, Mildner A, Varol D, Breker M, Strauss-Ayali D, Viukov S, Guilliams M, Misharin A, Hume DA, Perlman H, Malissen B, Zelzer E, Jung S (2013) Fate mapping reveals origins and dynamics of monocytes and tissue macrophages under homeostasis. Immunity 38:79–91. 10.1016/j.immuni.2012.12.001 23273845 PMC3908543

[B59] Zhang Y, Chen K, Sloan SA, Bennett ML, Scholze AR, Keeffe S, Phatnani HP, Guarnieri P, Caneda C, Ruderisch N, Deng S, Liddelow SA, Zhang C, Daneman R, Maniatis T, Barres BA, Wu JQ (2014) An RNA-sequencing transcriptome and splicing database of glia, neurons, and vascular cells of the cerebral cortex. J Neurosci 34:11929–11947. 10.1523/JNEUROSCI.1860-14.2014 25186741 PMC4152602

