# Supplementary figures and images for: Minimizing the Ex Vivo Confounds of Cell-Isolation Techniques on Transcriptomic and Translatomic Profiles of Purified Microglia
Source: eNeuro. 2022 Mar 28;9(2):ENEURO.0348-21.2022. doi: 10.1523/ENEURO.0348-21.2022 (PMC8970438; doi:10.1523/ENEURO.0348-21.2022)

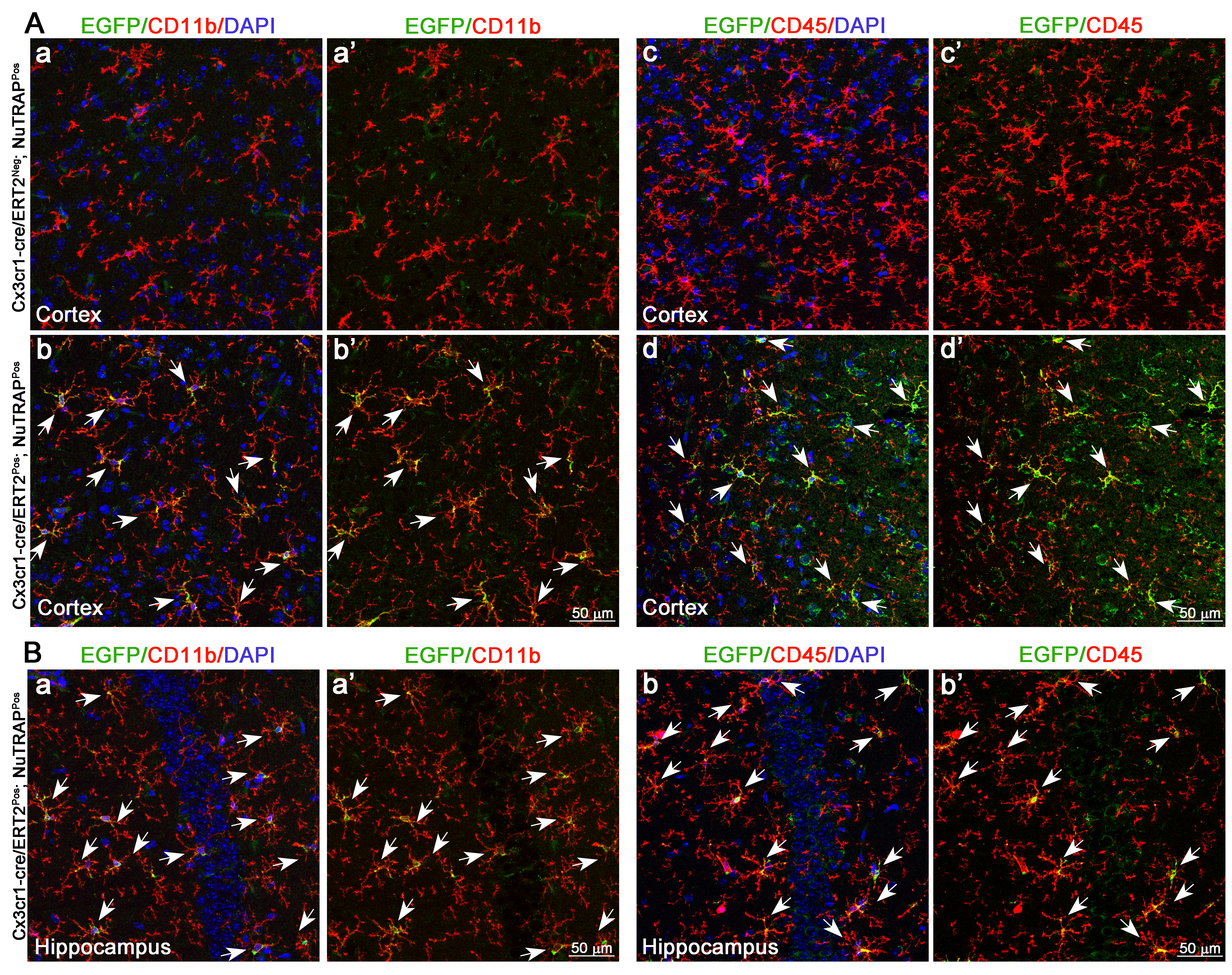

Supplement: Extended Data Figure 1-2 — Validation of microglial identity of recombined cells in the Cx3cr1-NuTRAP brain. Two months after Tam treatment, brains were harvested from Cx3cr1-NuTRAP and cre-negative NuTRAP+ (control) mice for immunohistochemistry (IHC). A, Representative confocal fluorescent microscopy images of sagittal brain sections captured in the cortex show eGFP expression (green signal) in cells that co-expressed CD11b (red signal, a, a’, b, b’) and CD45 (red signal, c, c’, d, d’) in Cx3cr1-NuTRAP brains but not in the cre-negative counterparts (n = 2/group). B, Representative confocal fluorescent microscopy images captured in the hippocampus show eGFP expression (green signal) in cells that co-expressed CD11b (a, a’) and CD45 (b, b’) in Cx3cr1-NuTRAP brains DAPI: nuclear counterstain. Scale bar: 50 μm Download Figure 1-2, TIF file. [file enu-eN-NWR-0348-21-s10.tif]
